# Polygenic Score for Conscientiousness Is a Protective Factor for Reversion from Mild Cognitive Impairment to Normal Cognition

**DOI:** 10.1002/advs.202309889

**Published:** 2024-06-05

**Authors:** Xuan Yang, Zirui Wang, Haonan Li, Wen Qin, Nana Liu, Zhixuan Liu, Siqi Wang, Jiayuan Xu, Junping Wang, for the Alzheimer's Disease Neuroimaging Initiative

**Affiliations:** ^1^ Department of Radiology Tianjin Key Lab of Functional Imaging & Tianjin Institute of Radiology Tianjin Medical University General Hospital Tianjin 300052 P. R. China; ^2^ Department of Radiology Jining No.1 People's Hospital Jining Shandong 272000 P. R. China

**Keywords:** alzheimer's disease, mild cognitive impairment, personality, polygenic score, reversion, structural covariance network

## Abstract

Spontaneous reversion from mild cognitive impairment (MCI) to normal cognition (NC) is little known. Based on the data of the Genetics of Personality Consortium and MCI participants from Alzheimer's Disease Neuroimaging Initiative, the authors investigate the effect of polygenic scores (PGS) for personality traits on the reversion of MCI to NC and its underlying neurobiology. PGS analysis reveals that PGS for conscientiousness (PGS‐C) is a protective factor that supports the reversion from MCI to NC. Gene ontology enrichment analysis and tissue‐specific enrichment analysis indicate that the protective effect of PGS‐C may be attributed to affecting the glutamatergic synapses of subcortical structures, such as hippocampus, amygdala, nucleus accumbens, and caudate nucleus. The structural covariance network (SCN) analysis suggests that the left whole hippocampus and its subfields, and the left whole amygdala and its subnuclei show significantly stronger covariance with several high‐cognition relevant brain regions in the MCI reverters compared to the stable MCI participants, which may help illustrate the underlying neural mechanism of the protective effect of PGS‐C.

## Graphical abstract

It is found that the polygenic score for conscientiousness is a protective factor for the reversion from mild cognitive impairment to normal cognition. This protective effect might be attributed to the influence of the polygenic score for conscientiousness on the subcortical structural glutamatergic synapses and structural connection between the left hippocampus and amygdala and higher cognitive brain regions.

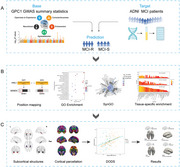

## 1 Introduction

Mild cognitive impairment (MCI) is a symptomatic predementia stage of Alzheimer's disease (AD) characterized by having an objective decline in cognition but reserving abilities for daily living.^[^ ^1^, ^2^ ^]^ Previous studies of MCI mainly focused on its higher risk of progression to dementia,^[^ ^3^, ^4^, ^5^, ^6^, ^7^ ^]^ However, the issue of subjects with MCI reverting to normal cognition (NC) has to date been taken in limited consideration; only several studies^[^ ^8^, ^9^, ^10^, ^11^ ^]^ indicated that MCI could also spontaneously revert to NC. Multiple demographic,^[^ ^12^ ^]^ clinical,^[^ ^13^ ^]^ psychological,^[^ ^14^ ^]^ and neuroimaging factors^[^ ^15^ ^]^ that may contribute to the reversion of MCI were evaluated in recent years. Among these factors, personality traits, which refer to fairly stable patterns to think, behave, and react, were preliminarily found to predict the reversion from MCI to NC.^[^ ^14^, ^16^ ^]^ The personality traits are organized by the widely accepted Five‐Factor Model, also known as the “Big Five”, into five dimensions: extraversion, agreeableness, conscientiousness, neuroticism, and openness to experience.^[^ ^17^ ^]^ There have been shown that MCI patients with lower neuroticism, higher extraversion^[^ ^14^ ^]^ or higher openness^[^ ^16^ ^]^ show an increased probability of reversion to NC, which could be used as predictors for reversion.

**Figure 1:**
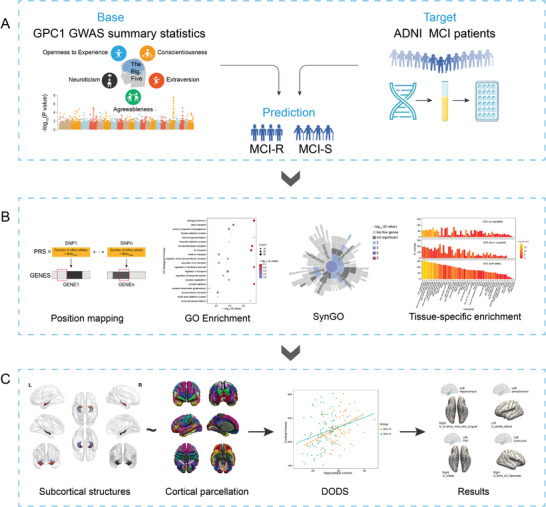
An overview of our study. A) Prediction effect of PGS for personality traits on the reversion from MCI to NC. B) SNPs included in the best PGS model were positionally mapped to genes, and gene enrichment analyses were performed. C) Seed‐based SCN analyses were performed with the volumes of subcortical structures as the seeds. Abbreviations: ADNI, Alzheimer's disease Neuroimaging Initiative; DODS, different offset, different slope; GO, gene ontology; GPC 1, Genetics of Personality Consortium phase 1; GWAS, genome‐wide association study; MCI, mild cognitive impairment; MCI‐R, mild cognitive impairment participants who reverted to normal cognition at year 1; MCI‐S, mild cognitive impairment participants who remained stable at year 1; NC, normal cognition; PGS, polygenic score; SCN, structural covariance network.

Twin studies have shown that personality traits are moderately heritable,^[^ ^18^ ^]^ and genome‐wide association studies (GWASs) have identified significant single nucleotide polymorphisms (SNPs) associated with personality traits.^[^ ^19^, ^20^, ^21^, ^22^ ^]^ Rather than just using SNPs that reach statistically genome‐wide significance, a recent approach namely polygenic score (PGS) is calculated by aggregating a much larger number of SNPs weighted by their effect size estimate,^[^ ^23^ ^]^ which can explain more variations for complex traits that would be hardly detected using single variant with small effect size.^[^ ^24^ ^]^ However, the role of the PGS for personality traits on the reversion from MCI to NC is still unknown. The first aim of this study was to evaluate the role of the PGS for personality traits on the reversion from MCI to NC. We selected PGS for personality traits rather than personality traits themselves since the former allows us to investigate the individual genetic predictions of personality traits and their underlying neurobiology. We then mapped the SNPs included in the best PGS model to genes based on genomic location and performed genes enrichment analysis to identify potential functions of these genes. We also performed tissue‐specific enrichment analysis to explore in which tissue these genes are significantly expressed. If the genes significantly expressed in the brain, we further investigated neuroanatomical substrates underlying the protective or adverse effect for reversion using structural covariance network (SCN) analysis. Structural covariance is a genetically heritable neuroimaging trait known as inter‐individual differences in the morphology (e.g., cortical volume, cortical thickness) of a brain region covary with other brain regions,^[^ ^25^ ^]^ which has been shown to partially reflect the underlying fiber connection^[^ ^26^ ^]^ and functional connectivity.^[^ ^27^ ^]^ Based on the brain regions with the most significant gene expression as seeds, we performed the seed‐based SCN analyses to compare the shared variation in cortical and subcortical volume, which might be one of the neuroimaging factors contributing to the reversion of MCI to NC. An overview of our study is summarized in **Figure** **1**.

## 2 Results

### 2.1 Demographics

A total of 395 participants (85 MCI reverters [MCI‐R], 310 stable MCI participants [MCI‐S]) were included in the PGS analysis, and 387 participants (84 in MCI‐R, 303 in MCI‐S) were included in the SCN analysis. No notable differences in age at baseline, sex, and educational years between MCI‐R and MCI‐S groups were observed, but a significantly smaller proportion of participants carrying the *APOE ε4* allele was found in the MCI‐R group compared to those in the MCI‐S group (*χ* ^2^ = 9.70, *P* = 0.002). The demographic characteristics of participants are shown in **Table** **1** (387 participants) and Table S1 (Supporting Information) (395 participants).

**Table 1:** Demographic characteristics of MCI‐R and MCI‐S (Values are shown as *n* (%) or mean (SD). The significant variable is shown in bold and italic. Abbreviations: *APOE*, Apolipoprotein E.

Characteristics	MCI‐R	MCI‐S	*t*/χ^2^	*P*
Age	74.26 (6.47)	73.73 (7.31)	*t* = 0.61	0.545
Male	49 (58.3%)	182 (60.1%)	*χ* ^2^ = 0.08	0.775
Educational years	15.77 (2.47)	15.95 (2.94)	*t* = −0.50	0.615
*APOE ε4*+	33 (39.3%)	177 (58.4%)	***χ^2^ = 9.70***	***0.002***

### 2.2 PGS for Conscientiousness Is a Protective Factor for the Reversion from MCI to NC

**Figure 2:**
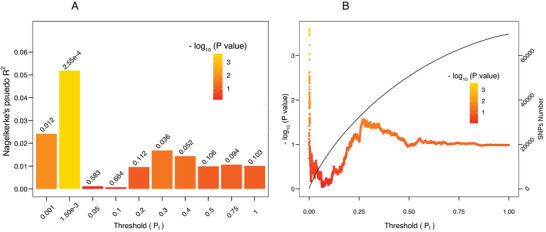
The predictability of PGS for conscientiousness on the reversion from MCI to NC. A) The bar plot shows the predictability of the best predictive model (*P*_t_ = 1.50e‐3) and other nine models constructed at broad *P*_t_ values (*P*_t_ = 0.001, 0.05, 0.1, 0.2, 0.3, 0.4, 0.5, 0.75, 1) on the reversion from MCI to NC. The color bar and the numbers above the bars show *P* values for the logistic regression between the PGS and reversion. The y‐axis shows variance explained by the PGS. B) The high‐resolution point plot presents the PGS models calculated at *P*_t_ from 5e‐8 to 1 with an increment of 5e‐5. The left y‐axis and the color bar show *P* values for the logistic regression between the PGS and reversion. The black line shows the number of SNPs included at different *P*_t_ (right y‐axis).

In the five personality traits, only PGS for conscientiousness (PGS‐C) was found to contribute to the reversion from MCI to NC (odds ratio [OR] = 1.19, 95% confidence interval [CI] = 1.08‐1.31, area under the curve = 0.6192 (Figure S1A, Supporting Information). The best model was at *P*_t_ = 1.50e‐3 with Nagelkerke's pseudo R^2^ of 5.59%, *P* value of 2.55e‐4, empirical *P* value of 9.60e‐3 and 486 SNPs included (**Figure** **2**). The MCI‐R group had a significantly higher PGS‐C compared to the MCI‐S group (*P* < 0.001) (Figure S1B, Supporting Information). We also found that the MCI participants carrying the *APOE ε4* allele have a significantly smaller chance of reversion than *APOE ε4* non‐carriers (OR = 0.85, 95% CI = 0.77‐0.94, *P* = 1.13e‐3). PGS for extraversion, agreeableness, neuroticism, and openness to experience were not found to affect the reversion from MCI to NC (Figures S2–S5 and Table S2, Supporting Information).

### 2.3 Gene Set Enrichment Analysis

**Figure 3:**
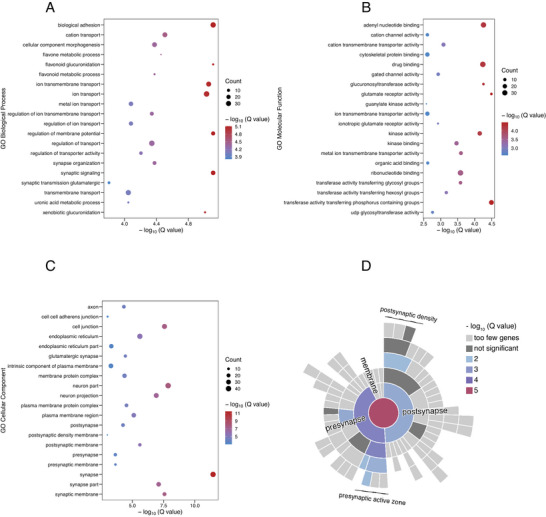
GO enrichment analyses. A–C) Top 20 enriched GO terms of BP, MF, and CC terms for positional mapped genes, respectively. The x‐axis and the color bars represent the ‐log_10_ (*q* value) of enrichment analysis. The y‐axis represents GO terms. The size of the spheres reflects the number of genes enriched in each item. D) The sunburst plots depict enriched CC terms of the synapse. Inner rings are parent terms of outer rings, and the color of each term is coded according to the enrichment ‐log_10_ (*q* value). Abbreviations: BP, biological process; CC, cellular component; MF, molecular function.

**Figure 4:**
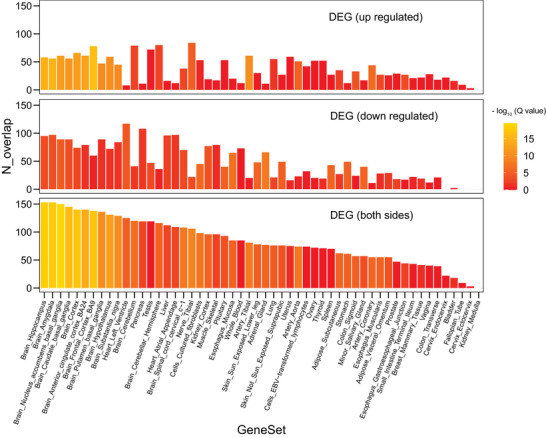
The bar plot shows the results of tissue‐specific enrichment analysis. The x‐axis represents 54 GTEx tissues. The y‐axis represents gene counts overlapped with the DEG set of each tissue. The color bar represents the enrichment ‐log_10_ (*q* value). Abbreviations: DEG, differentially expressed gene; GTEx, Genotype‐Tissue Expression.

Four hundred and eighty‐six SNPs included in the best PGS‐C model were mapped to 262 genes based on genomic location using MAGMA v1.10. Gene ontology (GO) enrichment analysis of these positional mapped genes was then performed using FUMA v1.3.8. The top 20 enriched biological process (BP), molecular function (MF), and cellular component (CC) terms were shown in **Figure** **3**A–C. The full lists of enriched items were shown in Table S3 (Supporting Information). The most enriched BP terms were synaptic signaling (false discovery rate [FDR] *q* = 7.75e‐6), regulation of membrane potential (FDR *q* = 7.75e‐6), synapse organization (FDR *q* = 4.21e‐5), and synaptic transmission, glutamatergic (FDR *q* = 1.57e‐4). The most enriched MF terms were glutamate receptor activity (FDR *q* = 3.23e‐5), gated channel activity (FDR *q* = 1.19e‐3), ionotropic glutamate receptor activity (FDR *q* = 1.20e‐3), and organic acid‐binding (FDR *q* = 2.42e‐3). The most enriched CC terms were related to the synapse, including synaptic membrane (FDR *q* = 2.68e‐8), postsynaptic membrane (FDR *q* = 2.51e‐6), glutamatergic synapse (FDR *q* = 3.63e‐5), and presynapse (FDR *q* = 5.60e‐5). In summary, these genes were mostly related to synapse and glutamatergic pathway. The tissue‐specific enrichment analysis results showed that these genes were significantly enriched for expression in diverse brain tissues (**Figure** **4**). More specifically, 153 of the 262 genes overlapped with the hippocampal DEG set, with the enrichment *q* value of 7.74e‐19, followed by the amygdala (153 overlapping genes, *q* value of 1.14e‐18), nucleus accumbens (150 overlapping genes, *q* value of 2.68e‐20), and caudate nucleus (145 overlapping genes, *q* value of 8.91e‐17). To further uncover which synaptic localizations and functions are mostly related to these genes, an enrichment analysis for this gene set was performed using SynGO. The enriched CC terms were presynapse (FDR *q* = 3.68e‐4), presynaptic active zone (FDR *q* = 3.82e‐4), presynaptic membrane (FDR *q* = 3.36e‐3), postsynapse (FDR *q* = 3.36e‐3), and postsynaptic density (FDR *q* = 8.75e‐3) (Figure 3D). The enriched BP terms were synapse organization (FDR *q* = 3.14e‐3), trans‐synaptic signaling (FDR *q* = 4.99e‐3), process in the presynapse (FDR *q* = 5.79e‐3), and regulation of the presynaptic membrane potential (FDR *q* = 3.14e‐3) (Figure S6, Supporting Information).

### 2.4 Seed‐Based SCN Analysis

**Figure 5:**
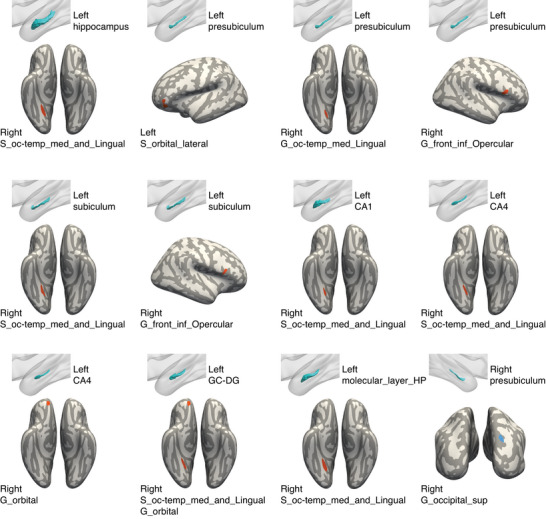
Differences in SCNs between the MCI‐R group and MCI‐S group with the volumes of hippocampus and its subfields as seeds. The left whole hippocampus and some of the left hippocampal subfields showed significantly stronger covariance with the right lingual gyrus, the right lingual sulcus, the right orbital gyri, the right opercular part of the inferior frontal gyrus and the left lateral orbital sulcus (all brain regions in red) in the MCI‐R group compared to the MCI‐S group. Only the right presubiculum showed significantly weaker covariance with the right superior occipital gyrus (in blue) in the MCI‐R group compared to the MCI‐S group (please see Table S4, Supporting Information, for detailed information). Abbreviations: GC‐DG, granule cell layer of the dentate gyrus; G_front_inf‐Opercular, opercular part of the inferior frontal gyrus; G_occipital_sup, superior occipital gyrus; G_oc‐temp_med‐Lingual, lingual gyrus, lingual part of the medial occipito‐temporal gyrus; G_orbital, orbital gyri; molecular_layer_HP, molecular layer of the hippocampus; S_oc‐temp_med_and_Lingual, medial occipito‐temporal sulcus (collateral sulcus) and lingual sulcus; S_orbital_lateral, lateral orbital sulcus.

**Figure 6:**
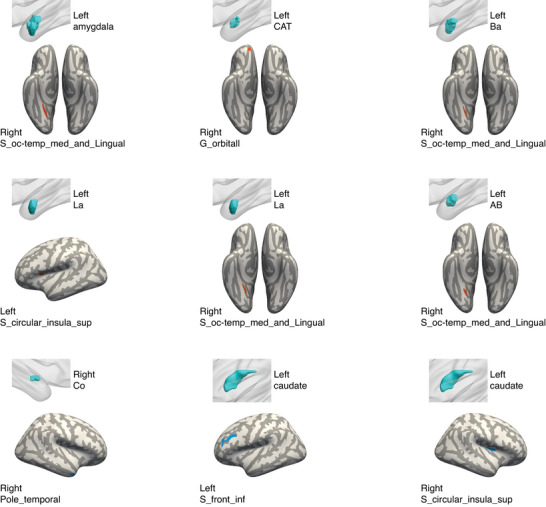
Differences in SCNs between the MCI‐R group and MCI‐S group with the volumes of amygdala and its subnuclei and caudate nucleus as seeds. The volumes of the left whole amygdala and some amygdala subnuclei showed significantly stronger covariance with the volumes of the right lingual sulcus, the left circular sulcus of the insula, and the right orbital gyri (all brain regions in red) in the MCI‐R group compared to the MCI‐S group. Only the volumes of the right amygdala cortical nucleus and left caudate nucleus showed significantly weaker covariance with volumes of several brain regions (in blue) in the MCI‐R group compared to the MCI‐S group (please see Table S4, Supporting Information for detailed information). Abbreviations: AB, accessory basal nucleus; Ba, basal nucleus; CAT, corticoamygdaloid transition area; Co, cortical nucleus; La, lateral nucleus; Pole_temporal, temporal pole; S_circular_insula_sup, superior segment of the circular sulcus of the insula; S_front_inf, inferior frontal sulcus.

The SCNs between the volumes of the whole hippocampus and some hippocampal subfields, the amygdala and some amygdala subnuclei, and left caudate nucleus, and the volumes of some brain regions showed significant differences between the MCI‐R group and MCI‐S group (please see **Figures** **5** and **6** and Table S4, Supporting Information, for detailed information). In general, the volumes of the left whole hippocampus and some of the left hippocampal subfields showed significantly stronger covariance with the volumes of the right lingual gyrus, the right lingual sulcus, the right orbital gyri, the right opercular part of the inferior frontal gyrus and the left lateral orbital sulcus in the MCI‐R group compared to the MCI‐S group. The volumes of the left whole amygdala and some amygdala subnuclei showed significantly stronger covariance with the volumes of the right lingual sulcus, the left circular sulcus of the insula, and the right orbital gyri in the MCI‐R group compared to the MCI‐S group. Only the volumes of the right hippocampal presubiculum, the right amygdala cortical nucleus, and left caudate nucleus showed significantly weaker covariance with volumes of several brain regions in the MCI‐R group compared to the MCI‐S group. No difference of structural covariance between the volumes of the nucleus accumbens and other brain regions was found in the MCI‐R group compared to the MCI‐S group.

### 2.5 Robustness Analysis

To validate the reproducibility of the results of SCN analyses, we used Schaefer 100 parcel, 7 network (Schaefer100_7), Schaefer200_7, and Schaefer400_7 atlases for cortical parcellation, and then re‐performed the SCN analyses. The results of the SCN analyses with the volumes of the hippocampus and its subfields, amygdala and its subnuclei, nucleus accumbens, and caudate nucleus as seeds were mostly consistent with the primary results, with only minor differences in *P* values (Figures S7–S12 and Tables S5–S7, Supporting Information). Only when using the Schaefer400_7 atlas for cortical parcellation, we failed to replicate the differences in covariance between the volumes of the left cortico amygdaloid transition area and the right amygdala cortical nucleus and the volume of cortex between the MCI‐R and MCI‐S groups.

## 3 Discussion

The present study assessed the association between the PGS for personality traits and the reversion from MCI to NC. Our results showed that PGS‐C could contribute to the reversion of MCI to NC. GO enrichment analysis suggested that these PGS related genes were mostly enriched in the synapse and glutamatergic pathway. Tissue‐specific enrichment analysis revealed that these genes were significantly enriched in diverse brain tissues, particularly in the hippocampus, amygdala, nucleus accumbens, and caudate nucleus. Finally, SCN analyses based on these gene‐enriched subcortical structures revealed that the volumes of the left whole hippocampus and some of its subfields, and the left whole amygdala and some of its subnuclei showed significantly stronger covariance with the volumes of some cognition‐relevant brain regions, such as the lingual gyrus and prefrontal cortex, in the MCI‐R group compared to the MCI‐S group, which might be the underlying neural mechanism of reversion.

The reversion from MCI to NC is relatively common in the most previous studies, with an overall reversion rate of approximately 20%−40%.^[^ ^8^, ^9^, ^10^, ^11^ ^]^ But the reversion rate of Alzheimer's Disease Neuroimaging Initiative (ADNI) cohort at one year of follow‐up was reported to be an extremely low 2.2%^[^ ^28^ ^]^ or 3%.^[^ ^29^ ^]^ Thomas et al.^[^ ^30^ ^]^ reported that the implementation of Jak/Bondi neuropsychological (NP) criteria in the ADNI cohort resulted in a more accurate reversion rate than the surprisingly low reversion rate reported in the previous studies using the conventional Peterson criteria. In the current study, we reclassified the cognitive status of all participants as NC, MCI or dementia at baseline and year 1 using Jak/Bondi NP criteria, and reported a reversion rate of 17%. The reclassified MCI‐R and MCI‐S groups were enrolled in the final analysis. In consideration of the lower dementia risk of MCI reverters than stable MCI participants,^[^ ^30^ ^]^ it is necessary to investigate the factors contributing to the reversion.

A meta‐analysis synthesized researches on the association between the personality and the risk of dementia and revealed that higher conscientiousness is associated with a lower risk of dementia.^[^ ^31^ ^]^ Additionally, Terracciano et al.^[^ ^32^ ^]^ examined the relationship between personality traits and amyloid and tau deposition, which are defining markers of AD neuropathology, and found that conscientiousness was a protective factor against AD key neuropathology. We reported here that the MCI‐R group had a higher PGS‐C compared to the MCI‐S group and the most importantly, the higher PGS‐C could contribute to the reversion from MCI to NC. Our findings were consistent with previous studies and suggested that higher PGS‐C is a potentially protective factor for MCI reversion. To our knowledge, only two studies have examined the role of personality traits on the reversion from MCI to NC and reported that lower neuroticism, higher extraversion^[^ ^14^ ^]^ or higher openness to experience^[^ ^16^ ^]^ were associated with an increased probability of reversion from MCI to NC. However, neither study found an association between conscientiousness and the reversion of MCI. The discrepancies between the results of previous studies and our study might be explained by the different criteria used to classify the cognitive states of the participants as NC or MCI between the above‐mentioned studies and ours. *APOE ε4* is a well‐established genetic risk factor for AD. Participants carrying *APOE ε4* allele showed a significantly smaller chance of reversion than *APOE ε4* non‐carriers in our study, which is in accordance with previous studies.^[^ ^12^, ^33^ ^]^

To better understand the underlying neurobiology of the protective effect of PGS‐C on reversion of MCI, we mapped the SNPs included in the best model to genes based on genomic location and performed gene set enrichment analyses. The GO enrichment analyses using FUMA and SynGO revealed that these genes are mostly related to synaptic signaling and synapse organization, particularly in glutamatergic synapses. Conspicuous synaptic loss is a well‐recognized hallmark of AD and is more correlated with cognitive impairments than the number of amyloid β plaques and neurofibrillary tangles.^[^ ^34^, ^35^, ^36^ ^]^ The larger neurons, which typically represent glutamatergic excitatory neurons, are especially vulnerable in AD.^[^ ^37^ ^]^ Glutamate is the primary excitatory neurotransmitter in the human brain, and glutamatergic pathways play a crucial role in memory formation, maintenance, as well as different forms of synaptic plasticity.^[^ ^38^ ^]^ The alterations in vesicular glutamate transporters,^[^ ^39^ ^]^ ionotropic glutamate receptors (iGluRs),^[^ ^40^ ^]^ and excitatory amino acid transporters,^[^ ^41^ ^]^ which are essential for glutamatergic synapses, have been observed in the brain in AD animal models and patients. Our findings suggest that these genes may impact the glutamatergic synapses, and eventually promote the recovery of cognition in MCI reverters. For example, *GRIN2A* and *GRIN2B* encode subunits of the N‐methyl‐D‐aspartate receptors, members of the family of iGluRs which are involved in synaptic plasticity, and therefore correlate with memory formation and other higher cognitive functions.^[^ ^42^ ^]^

The tissue‐specific enrichment analysis showed that these genes significantly enriched in diverse brain tissues, particularly in the subcortical structures, such as hippocampus, amygdala, nucleus accumbens, and caudate nucleus. For instance, *GRIN2A* and *GRIN2B* both overlapped with the up‐regulated DEG sets of hippocampus, amygdala, nucleus accumbens, and caudate nucleus. Our results are consistent with the previous studies that the hippocampus,^[^ ^43^ ^]^ amygdala,^[^ ^44^ ^]^ nucleus accumbens,^[^ ^45^ ^]^ and caudate nucleus^[^ ^45^ ^]^ are vulnerable brain regions in MCI patients. The combination of GO and tissue‐specific enrichment analyses indicate that these genes may conspicuously affect the glutamatergic synapse in these subcortical structures, as well as in other brain regions, and therefore affect cognitive performance.

The hippocampus is composed of a set of cytoarchitectonically and functionally heterogeneous subfields. For example, the CA1 and CA2/CA3/dentate gyrus support episodic memory in a complementary way,^[^ ^46^ ^]^ whereas the ventral subiculum is associated with spatial memory consolidation.^[^ ^47^ ^]^ Similarly, the amygdala is composed of several heterogeneous subnuclei.^[^ ^48^ ^]^ In humans, the amygdala is crucial in emotional and social cognitive processes; however, the specific functions of its various subnuclei remain unclear.^[^ ^48^ ^]^ Thus, analyzing the hippocampus and amygdala not exclusively as a whole entity but also on a subfield or subnuclei level might yield more specific insights. Emerging evidence indicates that a number of brain networks change during the progression of AD^[^ ^49^ ^]^ and are associated with cognitive decline.^[^ ^50^ ^]^ Therefore, we performed the SCN analyses between the volumes of the whole hippocampus and its subfields, amygdala and its subnuclei, nucleus accumbens, and caudate nucleus, and the whole cerebral cortex to investigate the probable change of connection between these subcortical structures and other brain regions. We observed significantly stronger covariances of the volumes of the left whole and subfields of hippocampus with the volumes of the right lingual gyrus, the right lingual sulcus, the right orbital gyri, the right opercular part of the inferior frontal gyrus and the left lateral orbital sulcus in the MCI‐R group compared to the MCI‐S group. We also observed significantly stronger covariances of the volumes of the left whole amygdala and some subnuclei with the volumes of the right lingual sulcus, the left circular sulcus of the insula, and the right orbital gyri in the MCI‐R group compared to the MCI‐S group. The lingual gyrus is associated with visual processing^[^ ^51^ ^]^ but also has been reported to contribute to episodic memory consolidation.^[^ ^52^ ^]^ We speculate that the stronger covariances between the volumes of the left whole hippocampus and its subfields, the left whole amygdala and its subnuclei, and the volume of the right lingual gyrus in the MCI‐R group may contribute to the recovery of memory function. The orbital gyri, the opercular part of the inferior frontal gyrus and the lateral orbital sulcus belong to the prefrontal cortex. The prefrontal cortex is associated with emotional, social, and motivational processes and plays an essential role in cognition, such as working memory, and decision‐making.^[^ ^53^ ^]^ The stronger covariance between the volumes of the left whole hippocampus and its subfields, and the left corticoamygdaloid transition area, and the volume of prefrontal cortex in the MCI‐R group may also contribute to the recovery of cognitive function and the reversion of MCI. The insula is heavily connected to a number of cortical and subcortical brain regions, which support a variety of sensory, emotional, motivational, and cognitive functions.^[^ ^54^ ^]^ The stronger covariance between the volumes of the left amygdala lateral nucleus and the left insula in the MCI‐R group may also contribute to the recovery of cognitive function as well as the reversion of MCI. We also observed the volumes of the right hippocampal presubiculum, the right amygdala cortical nucleus, and left caudate nucleus showed significantly weaker covariance with volumes of several brain regions in the MCI‐R group compared to the MCI‐S group. The neurobiological significance of this weaker structural covariance in the MCI‐R group compared to the MCI‐S group requires further investigation.

The results of SCN analyses based on Schaefer's parcellations were mostly consistent with the primary SCN analysis based on Destrieux atlas, demonstrating the good reproducibility of the SCN analyses in this study. We only failed to replicate two significant results of the primary SCN analyses using the volumes of two amygdala subnuclei as seeds based on Schaefer400_7 parcellation since different atlases delineating different regional boundaries with spatial discrepancies likely impact study results.^[^ ^55^ ^]^

To our knowledge, this is the first study to investigate the association between the PGS for personality and reversion from MCI to NC and we believe our findings provide some novel insights. However, there are some limitations in this study. First, our study only examined the reversion of MCI participants at one year of follow‐up, therefore the final reverters and stable MCI participants might be different due to the short initial observation period. Second, the sample size of target data in PGS analysis was small, which may induce bias, so larger sample size is needed in future studies. Third, we didn't validate the predictive ability of PGS‐C on conscientiousness because of the lack of personality information in the ADNI dataset. Fourth, gene set enrichment analysis is not directional. Therefore, further researches are needed to reveal the way that genes influence glutamatergic synapses. Last but not the least, our SCN analysis was performed at a group level, which did not allow the assessment of the association between individual PGS‐C with SCN.

## 4 Conclusion

In conclusion, PGS‐C is a potentially protective factor for reversion from MCI to NC. This protective effect may be attributed to affecting the subcortical structural glutamatergic synapses. The stronger structural connection between volumes of the left hippocampus and amygdala and the volumes of high‐cognition relevant brain regions in the MCI‐R group may help illustrate the underlying neural mechanism of the protective effect of PGS‐C.

## 5 Experimental Section

### Base Dataset

The PGS for personality analysis requires two datasets: base and target datasets. GWAS summary statistics for extraversion, agreeableness, conscientiousness, neuroticism, and openness to experience from the first phase of Genetics of Personality Consortium (GPC‐1)^[^ ^19^ ^]^ were used as base dataset. The GPC‐1 combined results of 10 GWASs for each Big Five personality trait, and all participants of GPC‐1 (*n* = 17 375) were of European ancestry.

### Target Dataset

The ADNI database (https://adni.loni.usc.edu) was used as target dataset. Participants with a diagnosis of NC and MCI at baseline in ADNI‐1, ADNI‐GO, and ADNI‐2 cohorts were enrolled in the study (total *n* = 1208). The cognitive states of all participants were reclassified as NC or MCI at baseline and reclassified as NC, MCI or dementia at year 1 based on Jak/Bondi NP criteria^[^ ^30^ ^]^ and ADNI's AD criteria.^[^ ^56^ ^]^ In brief, an MCI diagnosis would be made if an undemented participant meets any of the following three criteria: 1) performance > 1 SD below the demographically adjusted mean on both measures within any of three cognitive domains (i.e., language, memory, or attention/executive function); 2) performance > 1 SD below the demographically adjusted mean on at least one measure in each of three cognitive domains; 3) has a Functional Activities Questionnaire (FAQ) score larger than 5. Finally, 433 MCI patients at the baseline were included in the following analysis. Based on the 1‐year follow‐up results, MCI patients were divided into MCI‐R (*n* = 90) and MCI‐S (*n* = 343) groups. Detailed information about the base dataset and target dataset is provided in the Supporting Information.

### Genotype Data and Quality Control

Among 433 participants, 216 participants from the ADNI‐1 cohort were genotyped using the Illumina Human610‐Quad BeadChip, and 181 participants from ADNI‐GO/2 cohorts were genotyped using the Illumina HumanOmniExpress BeadChip.

In the sample‐level quality control (QC), participants with a genotyping rate <90%, possible relative relationship by using the estimate of pairwise identity‐by‐descent, and sex mismatch between genotyping and self‐reported data were excluded. One participant in ADNI‐1 and 1 participant in ADNI‐GO/2 were excluded. In variant‐level QC, SNPs with call rate < 85%, minor allele frequency (MAF) <0.01, Hardy‐Weinberg equilibrium (HWE) *P* < 1e‐6, and ambiguous strands were excluded. After the sample‐ and variant‐level QC, 215 participants with 550 834 variants in ADNI‐1 and 180 participants with 649 700 variants in ADNI‐GO/2 were included in the following imputation. The retained SNPs were pre‐phased by SHAPEIT2^[^ ^57^ ^]^ and imputed by IMPUTE2^[^ ^58^ ^]^ with 1000 Genomes Phase 3 as the reference panel. After imputation, the SNPs were filtered with imputed info > 0.8, MAF > 0.05, call rate > 99%, and HWE *P* < 1e‐6. Finally, 395 MCI patients (MCI‐R, *n* = 85; MCI‐S, *n* = 310) with 3 368 385 SNPs were included in the PGS calculation. To correct the population stratification, we performed the principal components analysis (PCA), and the first five principal components were used as covariates in the PGS analysis. Detailed information on genotyping, quality control, and imputation steps are provided in the Supporting Information.

### Calculation of Polygenic Scores for Personality Traits

PGS for each dimension of the personality traits was calculated using PRSice‐2 software.^[^ ^59^ ^]^ PRSice‐2 is a dedicated PGS program using the standard “C+T” method, which computes PGS based on a subset of clumped SNPs combined with different *P* value thresholds (*P*_t_).^[^ ^60^ ^]^ This study used the average score method to calculate the PGS, which calculates the score as the sum of effect allele counts observed multiplied by the summary statistic, divided by the number of alleles included in the PGS model. In the base dataset, a clumping distance of 250 kb, *r* ^2^ > 0.1, and 1000 Genomes phase 3 data set for Europeans as linkage disequilibrium reference were used for performing SNP clumping. In the target dataset, multiple PGS was calculated at *P* value thresholds from 5e‐8 to 1 with an increment of 5e‐5 for each dimension of the personality traits.

### Statistical Analysis—Demographic Data

All baseline demographic parameters for each group (MCI‐R and MCI‐S) were compared using Statistical Package for Social Sciences (SPSS) for Windows version 23 (IBM Corp., Armonk, NY, USA). Independent *t*‐test and *chi*‐square test were used to analyze differences in continuous variables (age at baseline, years of education) and categorical variables (sex, carrying *APOE ε4*), respectively.

### PGS for Personality Traits

The association between the PGS for personality traits and the reversion of MCI was assessed using logistic regression while adjusting for age at baseline, sex, years of education, the copy number of *APOE ε4*, and first five PCA components. Nagelkerke's pseudo *R* ^2^ and empirical *P* value (computed by repeating permutation 10 000 times) were used to evaluate the explained variance and overfitting of PGS, respectively. PGS with the empirical *P* value < 0.05 and highest model‐fit (Nagelkerke's pseudo *R* ^2^) was considered the best predictive model, and then used in subsequent analyses. The receiver operating characteristic curve (ROC) was constructed to obtain the area under the curve and evaluate the predictive value of PGS for personality traits on the reversion from MCI to NC.

### Genes Mapping and Gene Set Enrichment Analysis

SNPs included in the best PGS model were mapped to genes based on genomic location in a 5 kb window upstream and downstream using MAGMA v1.10 (Multi‐marker Analysis of GenoMic Annotation).^[^ ^61^ ^]^ GO enrichment analysis of these genes was then performed using GENE2FUNC module based on FUMA v1.3.8^[^ ^62^ ^]^ (Functional Mapping and Annotation of Genome‐Wide Association Studies). To further investigate the tissue‐specific expression of these gene sets, we also performed tissue‐specific enrichment analysis using FUMA. In brief, gene expressions of 54 tissues integrated into FUMA were obtained from the Genotype‐Tissue Expression (GTEx) project version 8.^[^ ^62^ ^]^ After normalization of gene expressions and a two‐sided Student's *t*‐test for each gene in one tissue against all others, a differentially expressed gene (DEG) set for every tissue was obtained. Input genes were then tested against each of the DEG sets by a hypergeometric test. More detailed information could be found in Watanabe et al's work.^[^ ^62^ ^]^ To further uncover which synaptic localizations and functions are mostly related to these genes, an enrichment analysis for this gene set was performed using SynGO,^[^ ^63^ ^]^ which is a database for an evidence‐based, expert‐curated resource for synapse function and gene enrichment. FDR Benjamini‐Hochberg was used to correct for multiple testing, with *q* value < 0.05 regarded as significant level.

### Structural Image Acquisition and Preprocessing

In ADNI‐1, sagittal 3D T1‐weighted images were acquired on 1.5T MR scanners for each participant, and one‐fourth of participants were also scanned on 3T scanners using the same MR scanning protocol. In ADNI‐GO/2, images were acquired only on 3T scanners using a similar protocol. Details of the ADNI MR protocol can be found on the Laboratory of Neuroimaging website (https://adni.loni.usc.edu). Images of 395 participants at baseline were preprocessed with the standard FreeSurfer v6.0.0 (https://surfer.nmr.mgh.harvard.edu) recon‐all pipeline. The major preprocessing steps include motion correction, intensity normalization, removal of non‐brain tissue, Talairach transformation, white and gray matter segmentation, spherical registration, and cortical parcellation by using the Destrieux atlas. The cortical volume maps were smoothed with a 10 mm full‐width half‐maximum Gaussian blurring kernel. Volumes of subcortical structures, such as the nucleus accumbens and caudate nucleus, were acquired through the FreeSurfer‐Aseg approach which was integrated within the recon‐all pipeline. Segmentation of hippocampal subfields was then performed using the HippocampalSubfields module of FreeSurfer v6.0.0.^[^ ^64^ ^]^ This module could automatically segment the left or right hippocampus into 12 subfields based on a statistical atlas built upon ultra‐high resolution (≈0.1 mm isotropic) ex vivo (magnetic resonance imaging) MRI data. The 12 hippocampal subfields include parasubiculum, presubiculum, subiculum, CA1, CA3, CA4, granule cell layer of the dentate gyrus, hippocampus‐amygdala‐transition‐area, fimbria, molecular layer of the hippocampus, hippocampal fissure, and hippocampal tail (Figure S13, Supporting Information). Segmentation of the subnuclei of the amygdala was performed using the HippocampalSubfieldsAndNucleiOfAmygdala module of FreeSurfer v7.1.1.^[^ ^48^ ^]^ This module could automatically segment the left or right amygdala into nine subnuclei (anterior amygdaloid, corticoamygdaloid transition area; basal, lateral, accessory basal, central, cortical, medial, and paralaminar subnuclei). The total intracranial volume (TIV), an important covariate for volumetric analyses, was also calculated during the preprocessing steps.

After all the preprocessing steps, the segmentation of pial and white surfaces and hippocampal subfields were carefully checked for any obvious errors using Freeview v2.0. One participant was excluded due to the failure of white matter segmentation. ComBat method, which could remove inter‐site technical variability while preserving biological variability, was used to harmonize the preprocessed brain images and the volume data of subcortical structures.^[^ ^65^ ^]^ Performing the harmonization needs at least 2 participants in one site, and 7 participants who could not meet the requirement were removed. Finally, 387 participants were included in the following SCN analysis.

### Seed‐Based SCN Analysis

In the current study, the SCNs between the volumes of the seeds and the cortical volumes of the left or right hemisphere derived from baseline images were constructed in FreeSurfer v6.0.0., with the volumes of the whole hippocampus and its subfields, amygdala and its subnuclei, nucleus accumbens, and caudate nucleus as seeds. The differences in networks between the MCI‐R group and the MCI‐S group were then assessed using generalized linear models with the different offset different slope (DODS) model. A linear model implies two parameters: an offset (also called intercept) and a slope. The DODS model uses a separate linear model for each group (MCI‐R or MCI‐S), meaning that each group has its own offset and its own slope. Here, we opted for the DODS model because we hypothesized that the MCI‐R and MCI‐S groups might exhibit distinct rates of cortical volume changes. Detailed information on the DODS model is provided in the Supporting Information. The age at baseline, sex, years of education, the copy number of *APOE ε4*, and TIV were included as covariates. A threshold of *P* < 0.001 was used for cluster forming for the vertex‐wise analyses. Multiple comparisons were corrected using a cluster‐wise Monte Carlo simulation with 10 000 iterations, and the corrected cluster‐wise *P* value < 0.05 was regarded as significant.

### Robustness Analysis

Based on resting‐state functional MRI data, Schaefer et al.^[^ ^66^ ^]^ proposed a gradient‐weighted Markov RandomField model integrating local gradient and global similarity approach, and subsequently created a set of cortical parcellations at varying resolutions from 100 to 1000 regions of interest. Multiresolution Schaefer's parcellations are available at: https://github.com/ThomasYeoLab/CBIG/tree/master/stable_projects/brain_parcellation/Schaefer2018_LocalGlobal. In this study, we selected the commonly used Schaefer100_7, Schaefer200_7, and Schaefer400_7 atlases for cortical parcellation and re‐performed the SCN analyses. The parameters for SCN analyses were the same as those in the main analysis.

### Ethics Approval and Consent to Participate

ADNI was approved by the institutional review boards at each of the participating institutions, and written informed consent was obtained from all participants or authorized representatives at each site.

## Conflict of Interest

The authors declare no conflict of interest.

## Author Contributions

X.Y. and Z.W. contributed equally to this work. J.W., J.X., X.Y., and Z.W. designed the study. X.Y., Z.W., W.Q., N.L., Z.L., S.W., and H.L. analyzed the data. J.W., J.X., X.Y., and Z.W. wrote the manuscript. All authors critically reviewed the manuscript. Data used in preparation of this article were obtained from the Alzheimer's Disease Neuroimaging Initiative (ADNI) database (https://adni.loni.usc.edu). As such, the investigators within the ADNI contributed to the design and implementation of ADNI and/or provided data but did not participate in analysis or writing of this report. A complete listing of ADNI investigators can be found at: http://adni.loni.usc.edu/wp‐content/uploads/how_to_apply/ADNI_Acknowledgement_List.pdf.

## Supporting information

Supporting Information

Supporting Table 3

## Data Availability

The data that support the findings of this study are available on request from the corresponding author. The data are not publicly available due to privacy or ethical restrictions.
