# Supplementary material for: Polygenic Score for Conscientiousness Is a Protective Factor for Reversion from Mild Cognitive Impairment to Normal Cognition
Source: Adv Sci (Weinh). 2024 Jun 5;11(29):2309889. doi: 10.1002/advs.202309889 (PMC11304237; doi:10.1002/advs.202309889)
Supplement: Supplementary file 1 — Supporting Information [file ADVS-11-2309889-s001.docx]

**Supplementary Material**

**Contents:**

1. **Supplementary Figures S1-S13**

**Figure S1.** The predictive value of PGS-C on the reversion of MCI to NC

**Figure S2.** The predictive effect of PGS for extraversion on the reversion from MCI to NC

**Figure S3.** The predictive effect of PGS for agreeableness on the reversion from MCI to NC

**Figure S4.** The predictive effect of PGS for neuroticism on the reversion from MCI to NC

**Figure S5.** The predictive effect of PGS for openness to experience on the reversion from MCI to NC

**Figure S6.** Results of GO enrichment analysis using SynGO

**Figure S7.** Differences in SCNs (based on Schaefer100_7 parcellation) with the volumes of hippocampus and its subfields as seeds between the MCI-R group and MCI-S group.

**Figure S8.** Differences in SCNs (based on Schaefer100_7 parcellation) with the volumes of amygdala and its subnuclei and caudate nucleus as seeds between the MCI-R group and MCI-S group.

**Figure S9.** Differences in SCNs (based on Schaefer200_7 parcellation) with the volumes of hippocampus and its subfields as seeds between the MCI-R group and MCI-S group.

**Figure S10.** Differences in SCNs (based on Schaefer200_7 parcellation) with the volumes of amygdala and its subnuclei and caudate nucleus as seeds between the MCI-R group and MCI-S group.

**Figure S11.** Differences in SCNs (based on Schaefer400_7 parcellation) with the volumes of hippocampus and its subfields as seeds between the MCI-R group and MCI-S group.

**Figure S12.** Differences in SCNs (based on Schaefer400_7 parcellation) with the volumes of amygdala and its subnuclei and caudate nucleus as seeds between the MCI-R group and MCI-S group.

**Figure S13.** The spatial distribution of the 12 hippocampal subfields

1. **Supplementary Tables S1-7**

**Table S1.** Characteristics of MCI-R and MCI-S groups

**Table S2.** The best predictive model for the other 4 personality traits

**Table S3.** Results of GO enrichment analysis using FUMA

**Table S4.** Results of Seed-based SCN analysis (Destrieux atlas)

**Table S5.** Results of Seed-based SCN analysis (Schaefer100_7)

**Table S6.** Results of Seed-based SCN analysis (Schaefer200_7)

**Table S7.** Results of Seed-based SCN analysis (Schaefer400_7)

1. **Supplementary Methods**

**Base dataset**

**Target dataset**

**Genotyping and Quality control (QC) for ADNI-1 and ADNI-GO/2**

**Imputation and following QC**

**Different offset different slope (DODS) model**

1. **References**

**Supplementary Figures**


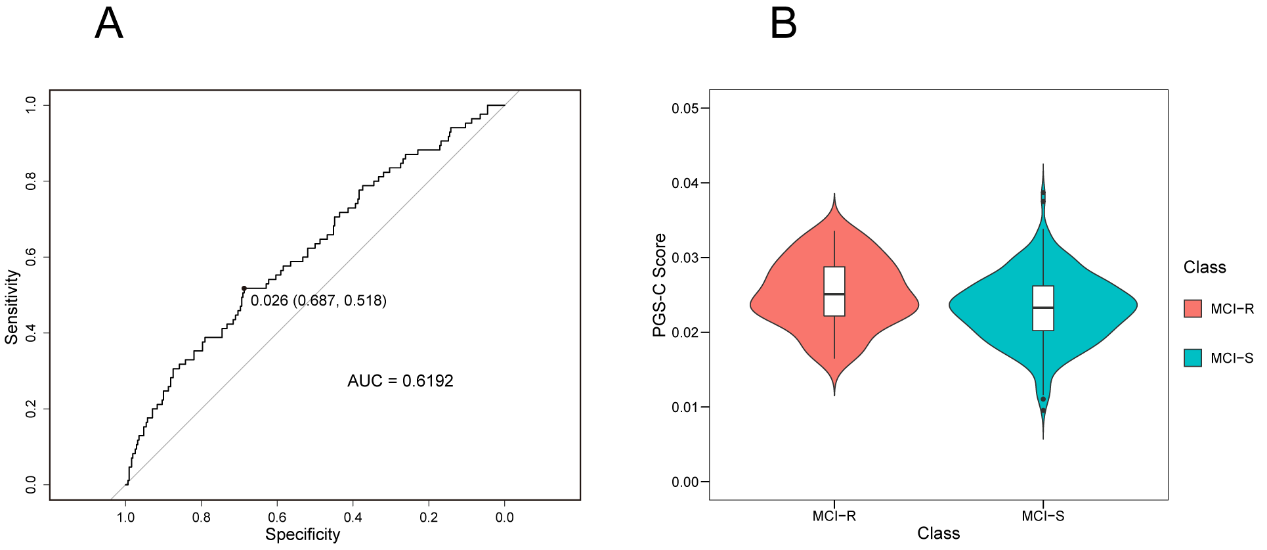


Figure S1. The predictive value of PGS-C on the reversion of MCI to NC. (A) The ROC depicts the sensitivity and specificity of the PGS-C in predicting the reversion from MCI to NC. (B) The violin plot indicates that the MCI-R group had a significantly higher PGS-C score than the MCI-S group.

Abbreviations: AUC, area under the curve; PGS-C, polygenic score for conscientiousness; ROC, receiver operating characteristic curve.


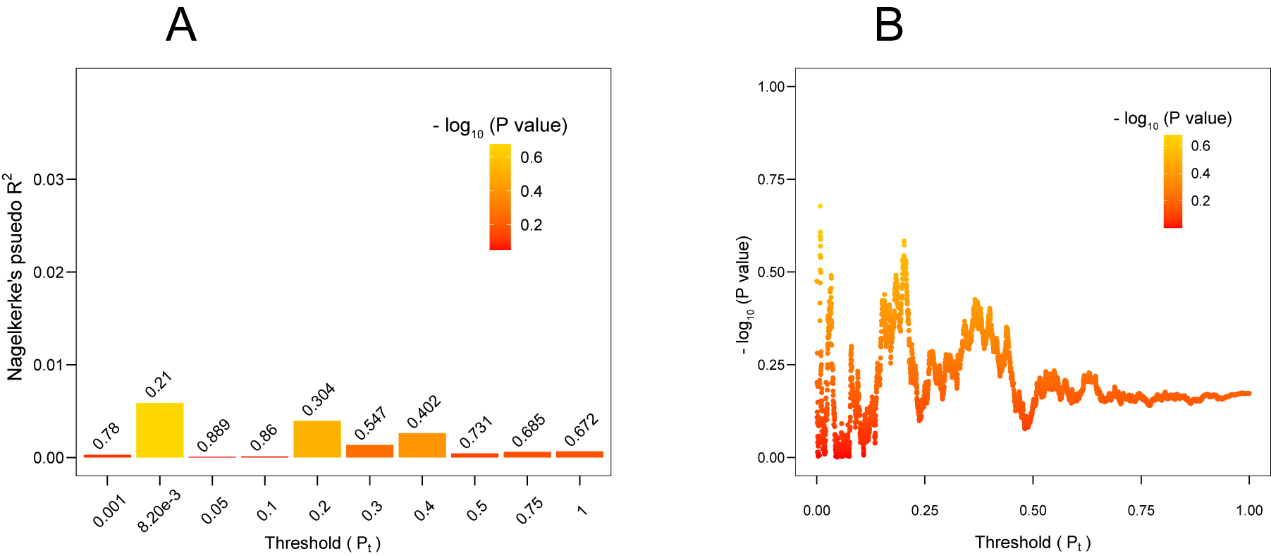


Figure S2. The bar plot and high-resolution point plot show the predictive effect of PGS for extraversion on the reversion from MCI to NC.


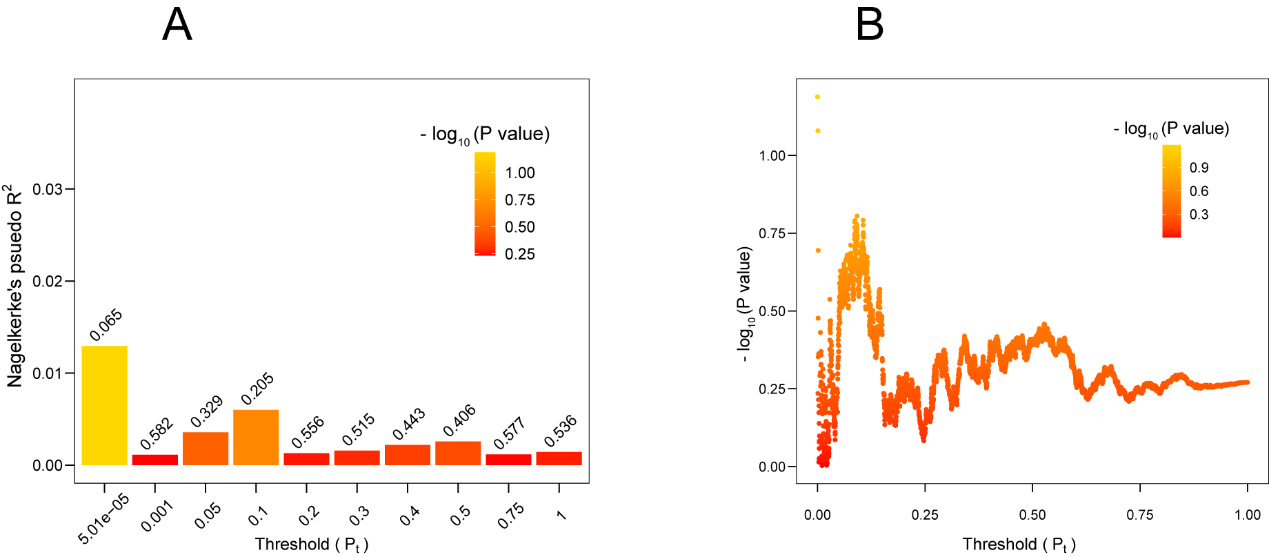


Figure S3. The bar plot and high-resolution point plot show the predictive effect of PGS for agreeableness on the reversion from MCI to NC.


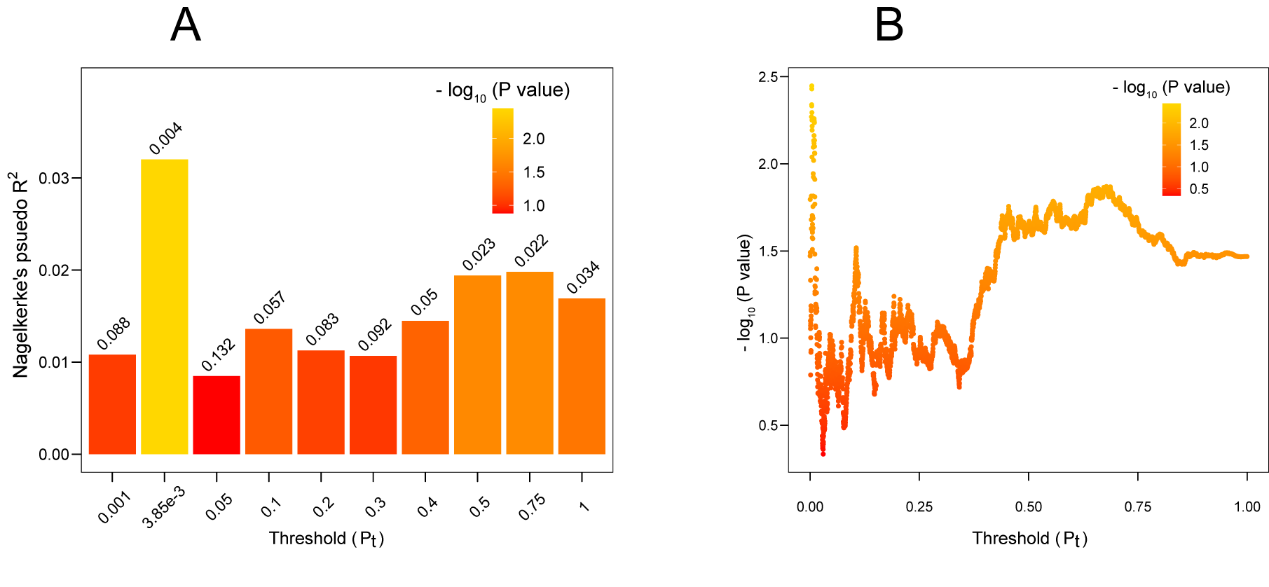


Figure S4. The bar plot and high-resolution point plot show the predictive effect of PGS for neuroticism on the reversion from MCI to NC. For neuroticism, the best predictive model was at *P*_t_ = 3.85e-3, with the *P* value of 3.57e-3, while the empirical *P* value was 0.067.


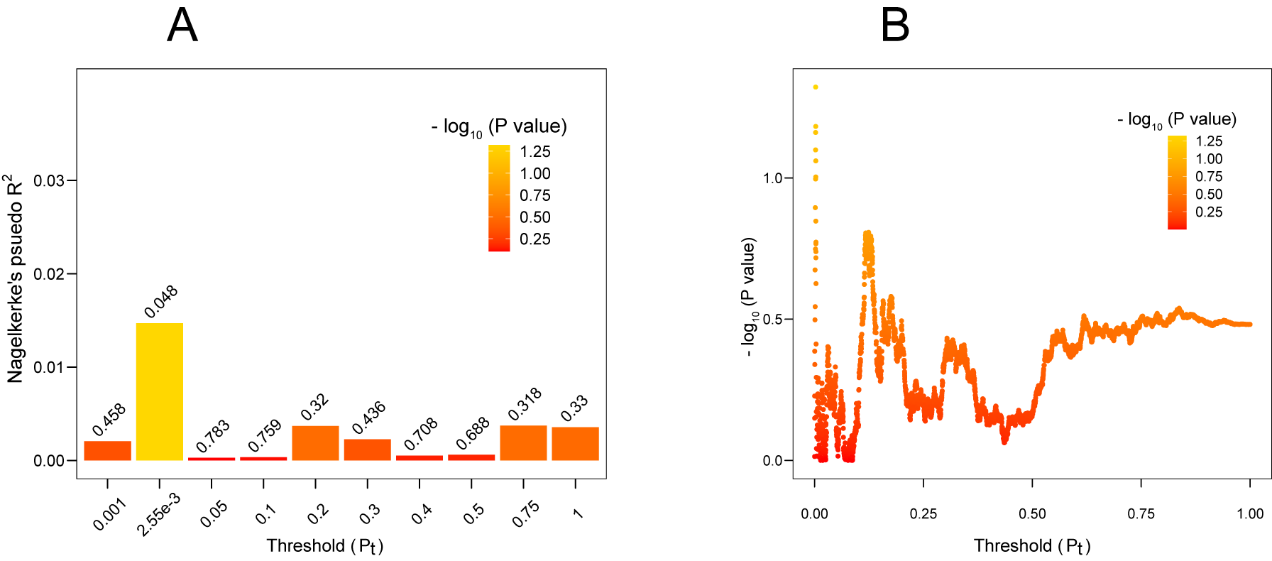


Figure S5. The bar plot and high-resolution point plot show the predictive effect of PGS for openness to experience on the reversion from MCI to NC. For openness to experience, the best predictive model was at *P*_t_ =2.55e-3, with the *P* value of 0.048, whereas the empirical *P* value was 0.477.


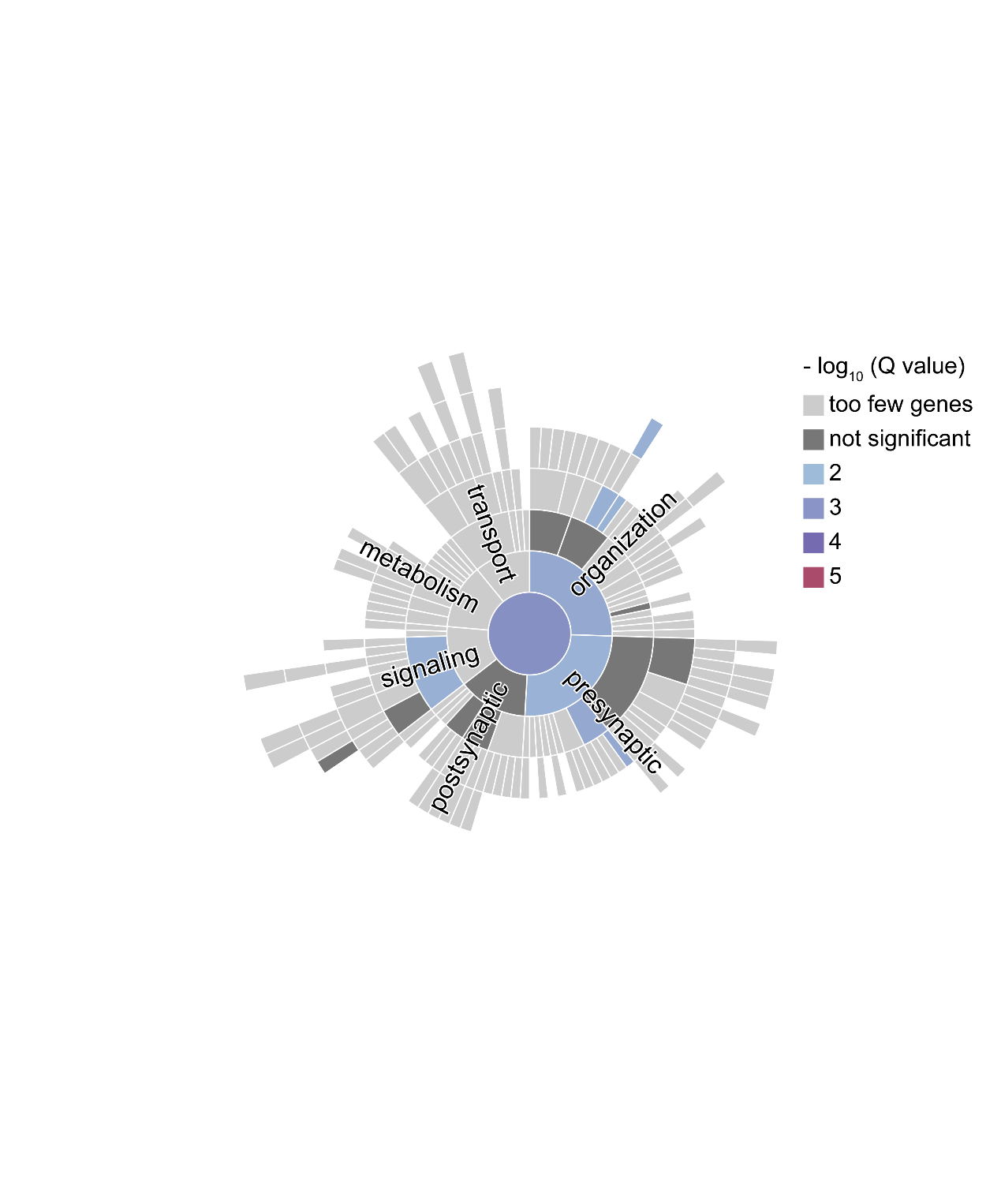


Figure S6. GO enrichment analysis using SynGO. The sunburst plots depict enriched BP terms of the synapse. Inner rings are parent terms of outer rings, and the color of each term is coded according to the enrichment -log_10_ (*q* value).

Abbreviations: GO, gene ontology; BP, biological process.


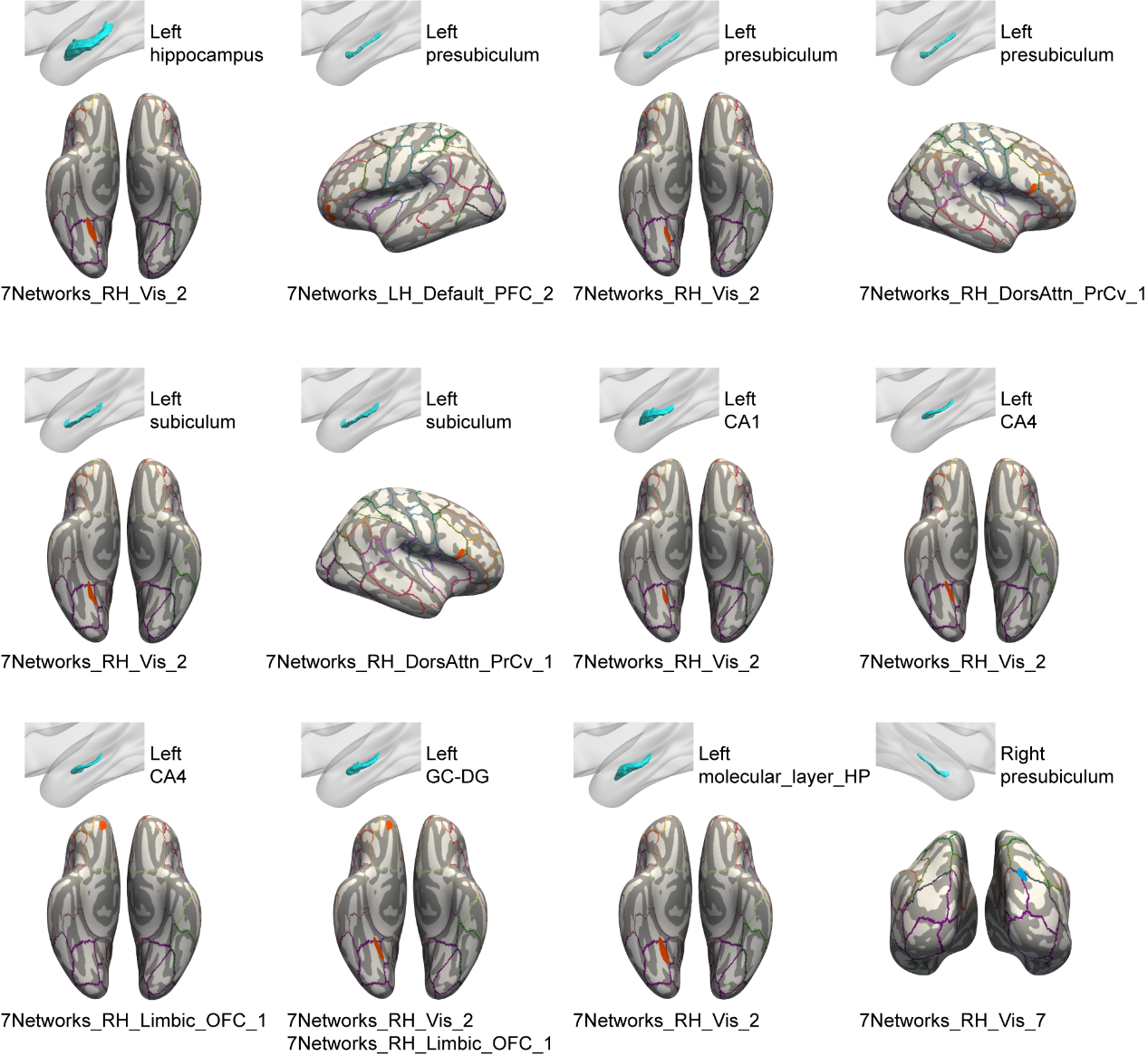


Figure S7. Differences in SCNs (based on Schaefer100_7 parcellation) with the volumes of hippocampus and its subfields as seeds between the MCI-R group and MCI-S group.

Abbreviations: DorsAttn, dorsal attention; GC-DG, granule cell layer of the dentate gyrus; LH, left hemisphere; molecular_layer_HP, molecular layer of the hippocampus; OFC, orbital frontal cortex; PFC, prefrontal cortex; PrCv, precentral ventral; RH, right hemisphere; Vis, visual.


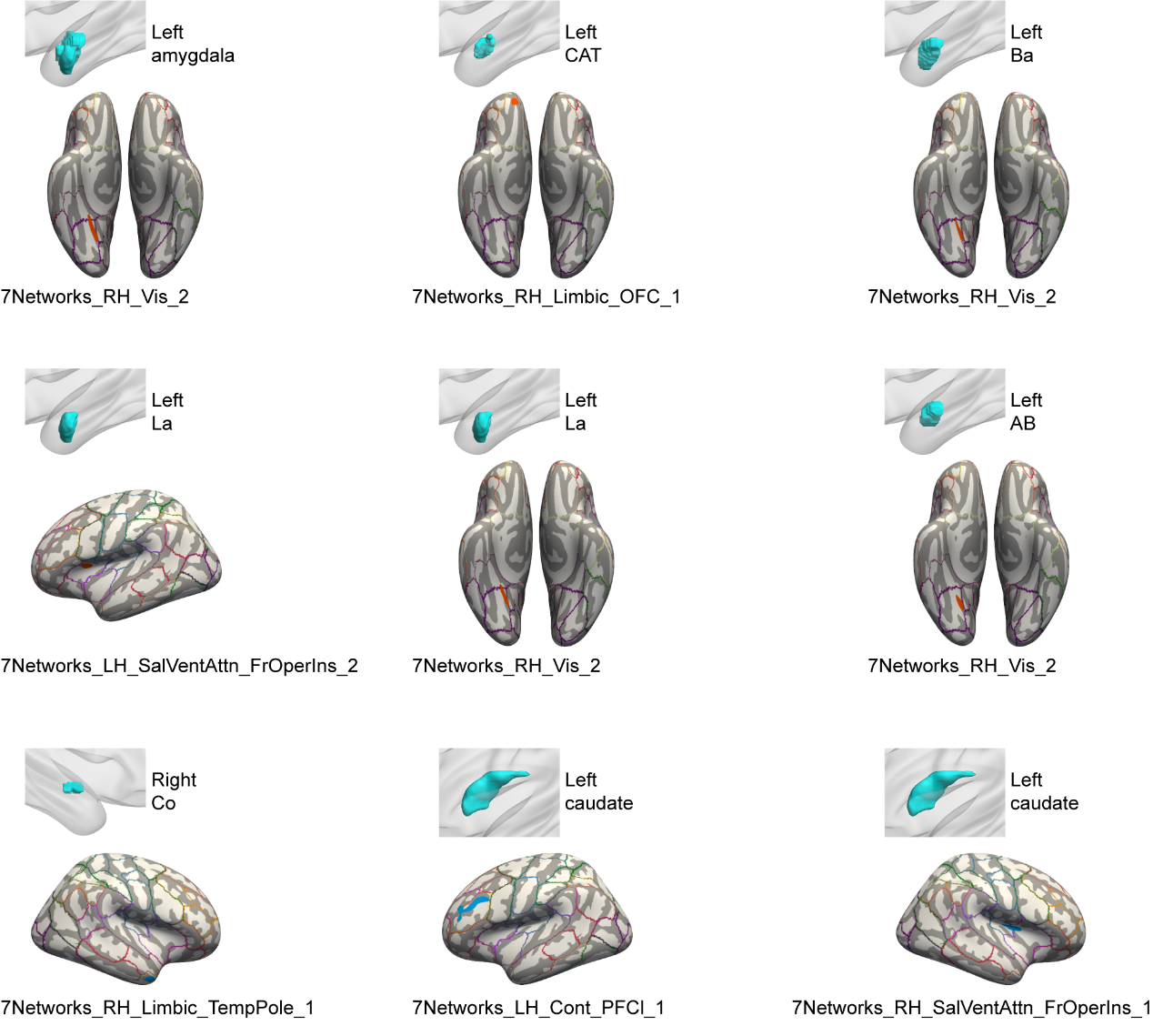


Figure S8. Differences in SCNs (based on Schaefer100_7 parcellation) with the volumes of amygdala and its subnuclei and caudate nucleus as seeds between the MCI-R group and MCI-S group.

Abbreviations: AB, accessory basal nucleus; Ba, basal nucleus; CAT, corticoamygdaloid transition area; Co, cortical nucleus; Cont, control; FrOperIns, frontal operculum insula; La, lateral nucleus; PFCl, lateral prefrontal cortex; SalVentAttn, salience / ventral attention; TempPole, temporal pole.


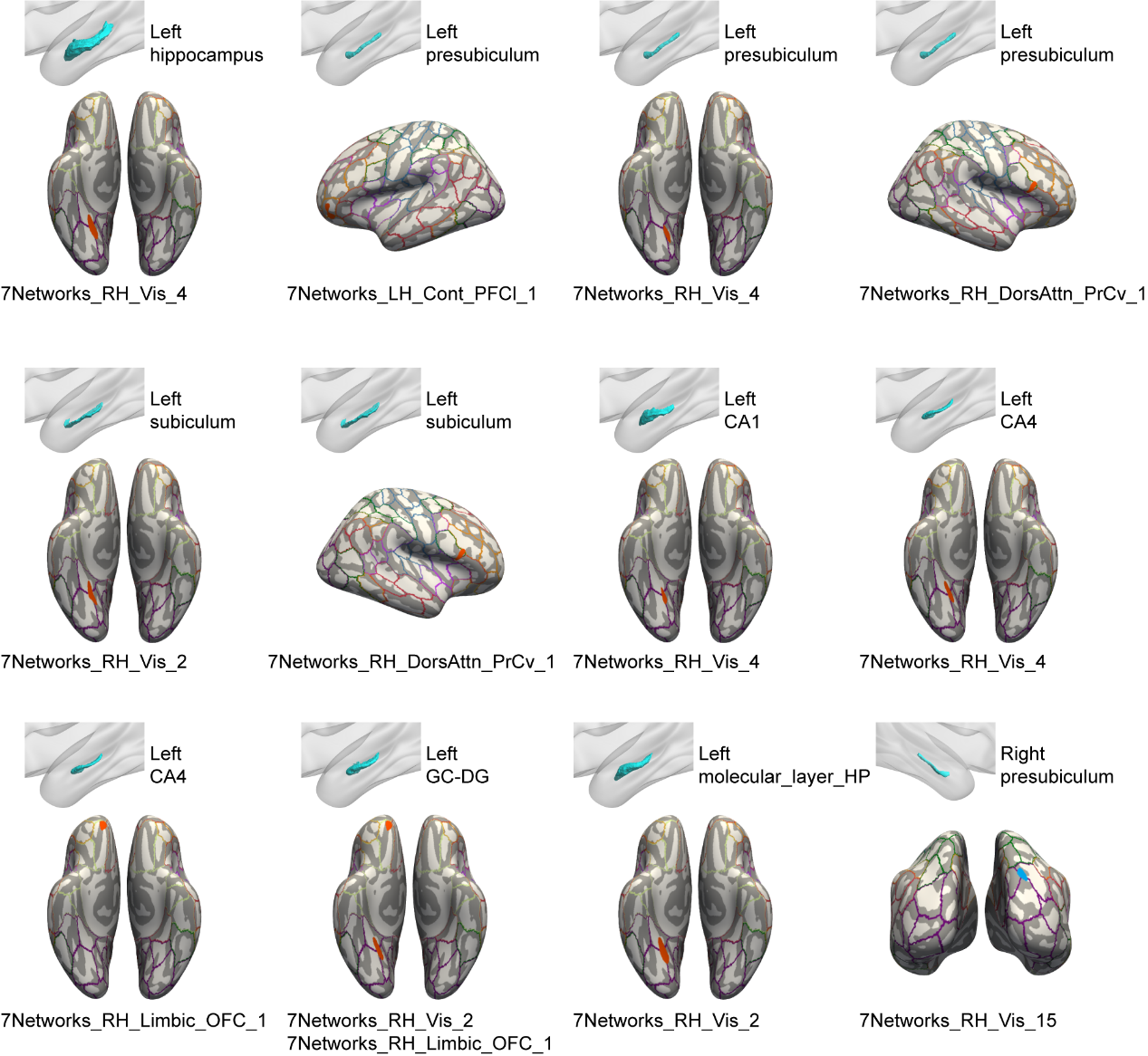


Figure S9. Differences in SCNs (based on Schaefer200_7 parcellation) with the volumes of hippocampus and its subfields as seeds between the MCI-R group and MCI-S group.


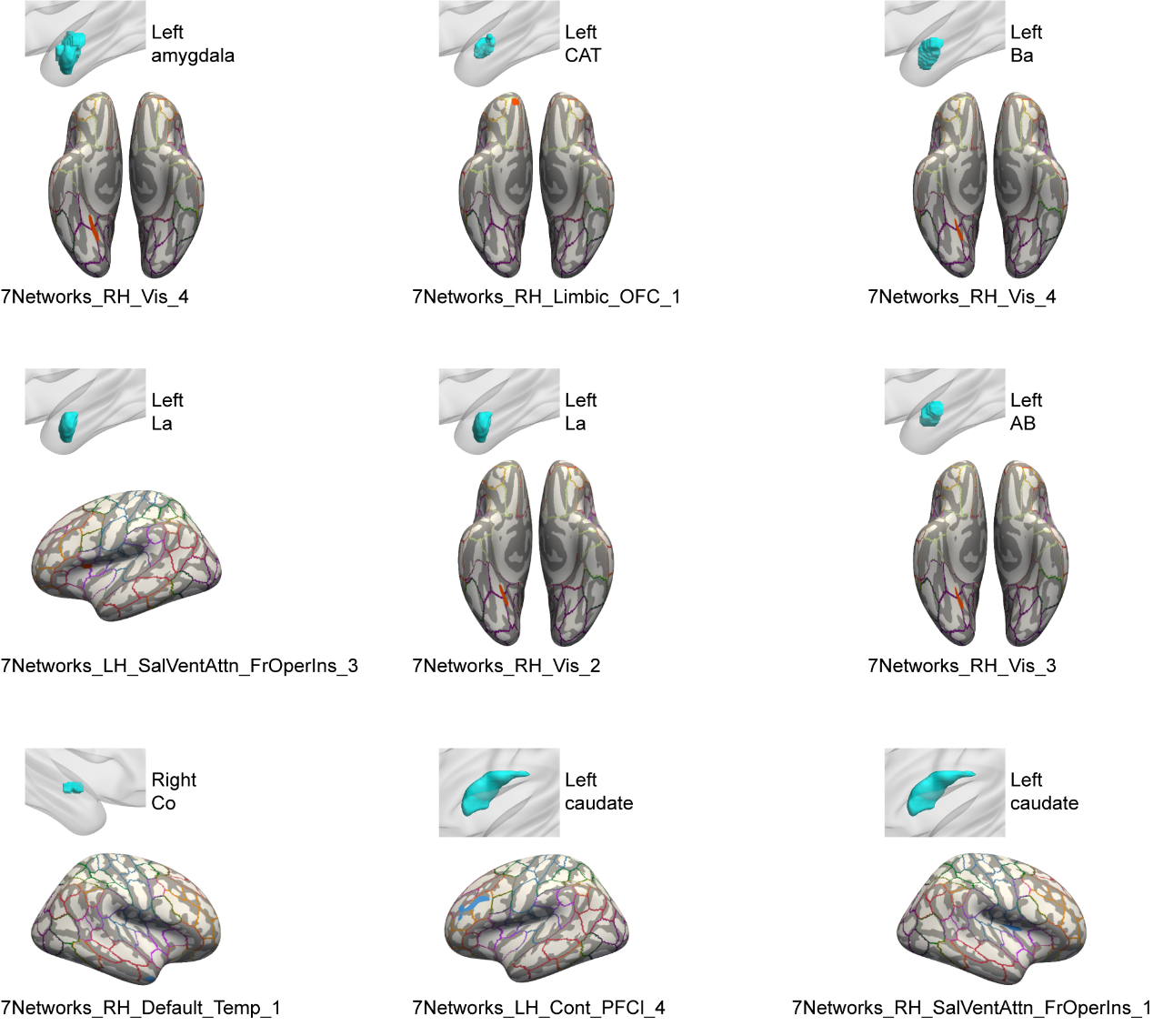


Figure S10. Differences in SCNs (based on Schaefer200_7 parcellation) with the volumes of amygdala and its subnuclei and caudate nucleus as seeds between the MCI-R group and MCI-S group.

Abbreviations: Temp, temporal.


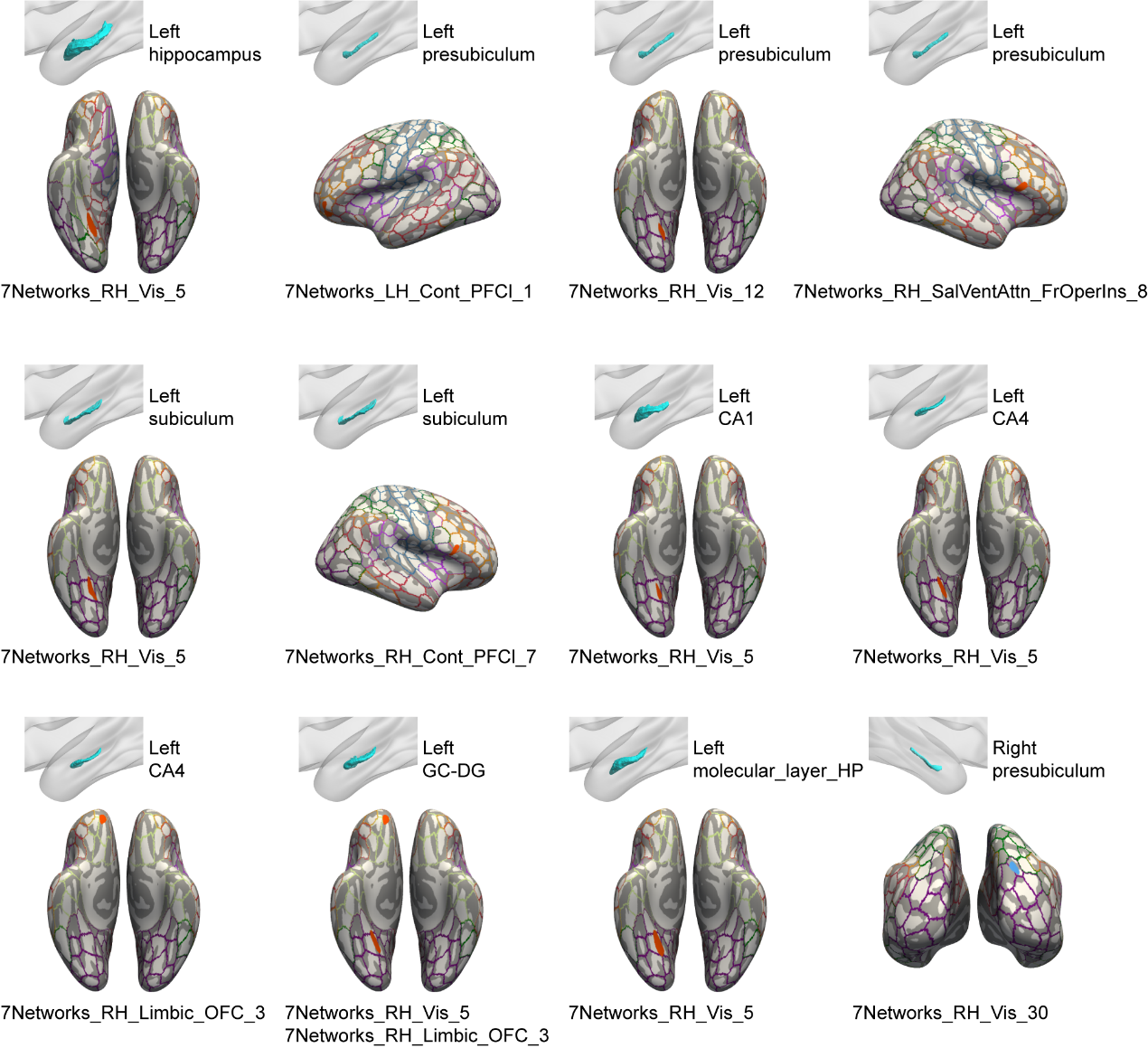


Figure S11. Differences in SCNs (based on Schaefer400_7 parcellation) with the volumes of hippocampus and its subfields as seeds between the MCI-R group and MCI-S group.


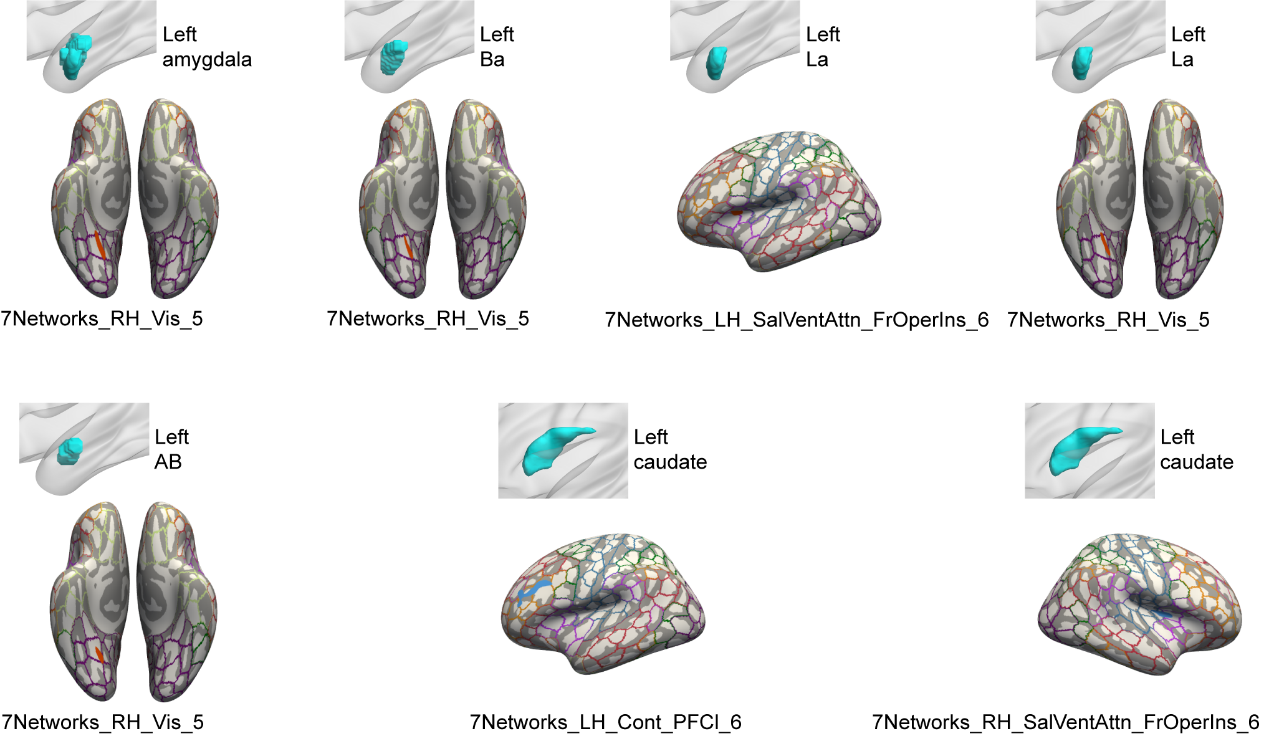


Figure S12. Differences in SCNs (based on Schaefer400_7 parcellation) with the volumes of amygdala and its subnuclei and caudate nucleus as seeds between the MCI-R group and MCI-S group.


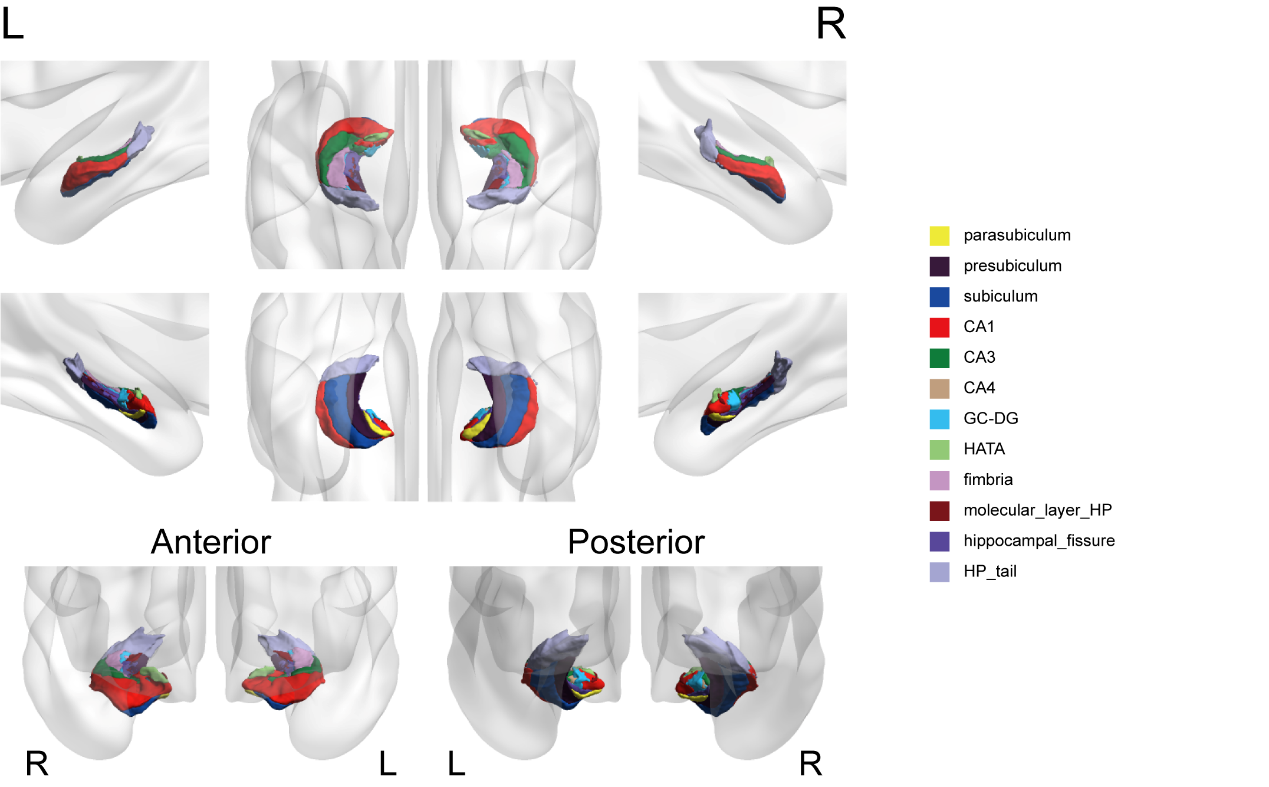


Figure S13. The spatial distribution of the 12 hippocampal subfields.

Abbreviations: HATA, hippocampus-amygdala-transition-area; HP_tail, hippocampal tail.

**Supplementary Tables**

**Table S1.** **Characteristics of MCI-R and MCI-S groups**

| Characteristics | MCI-R | MCI-S | t, χ^2^ | P |
| --- | --- | --- | --- | --- |
| Age | 74.15 (6.51) | 73.73 (7.29) | t = 0.49 | 0.626 |
| Male | 50 (58.8) | 186 (60.0) | χ^2^ = 0.04 | 0.845 |
| Education | 15.78 (2.45) | 15.95 (2.92) | t = -0.52 | 0.607 |
| PGS for Conscientiousness | 0.26 (0.05) | 0.24 (0.05) | t = 3.67 | 0.000282 |
| *APOE ε4*+ | 33 (38.8) | 180 (58.1) | ***χ^2^ = 9.94*** | ***0.002*** |

Values are shown as n (%) or mean (SD). The significant variable is shown in bold and italic.

Abbreviations: *APOE,* Apolipoprotein E; MCI-R, mild cognitive impairment participants who reverted to normal cognition at year 1; MCI-S, mild cognitive impairment participants who remained stable at year 1; PGS, polygenic score.

**Table S2.** **The best predictive model for the other 4 personality traits**

| Personality traits | Threshold | Pseudo R^2^ | OR (95% CI) | P value | Empirical P | Num_SNP |
| --- | --- | --- | --- | --- | --- | --- |
| Extraversion | 8.20e-3 | 0.63% | 0.94 (0.85-1.04) | 0.210 | 0.924 | 51 |
| Agreeableness | 5.01e-5 | 1.40% | 1.04 (1.00-1.08) | 0.065 | 0.557 | 1 |
| Neuroticism | 3.85e-3 | 3.45% | 0.86 (0.77-0.95) | 3.57e-3 | 0.067 | 29 |
| Openness to experience | 2.55e-3 | 1.59% | 0.90 (0.82-1.00) | 0.048 | 0.477 | 14 |

Abbreviations: CI, confidence interval; Num_SNP, number of SNPs; OR, odds ratio.

**Table S3. Results of GO enrichment analysis using FUMA**

**(See accompanying Excel file)**

**Table S4.** **Results of Seed-based SCN analysis (Destrieux atlas)**

| Seed | Different covariance brain regions | *Z* score | *P* value |
| --- | --- | --- | --- |
| Left whole hippocampus | Right S_oc-temp_med_and_Lingual | 4.701 | 0.0001 |
| Left presubiculum | Left S_orbital_lateral | 4.626 | 0.0002 |
|  | Right G_oc-temp_med-Lingual | 4.274 | 0.0001 |
|  | Right G_front_inf-Opercular | 4.763 | 0.0001 |
| Left subiculum | Right S_oc-temp_med_and_Lingual | 3.951 | 0.0001 |
|  | Right G_front_inf-Opercular | 3.544 | 0.0049 |
| Left CA1 | Right S_oc-temp_med_and_Lingual | 3.484 | 0.0032 |
| Left CA4 | Right S_oc-temp_med_and_Lingual | 4.379 | 0.0002 |
|  | Right G_orbital | 5.018 | 0.0129 |
| Left GC-DG | Right S_oc-temp_med_and_Lingual | 4.587 | 0.0002 |
|  | Right G_orbital | 4.941 | 0.0132 |
| Left molecular_layer_HP | Right S_oc-temp_med_and_Lingual | 4.994 | 0.0001 |
| Right presubiculum | Right G_occipital_sup | -3.739 | 0.0026 |
| Left whole amygdala | Right S_oc-temp_med_and_Lingual | 4.150 | 0.0001 |
| Left corticoamygdaloid transition area | Right G_orbital | 4.337 | 0.0319 |
| Left basal nucleus | Right S_oc-temp_med_and_Lingual | 3.680 | 0.0014 |
| Left lateral nucleus | Left S_circular_insula_sup | 3.916 | 0.0378 |
|  | Right S_oc-temp_med_and_Lingual | 3.935 | 0.0007 |
| Left accessory basal nucleus | Right S_oc-temp_med_and_Lingual | 3.889 | 0.0026 |
| Right cortical nucleus | Right Pole_temporal | -4.630 | 0.0110 |
| Left caudate nucleus | Left S_front_inf | -3.970 | 0.0001 |
|  | Right S_circular_insula_sup | -4.451 | 0.0095 |

Abbreviations: G_front_inf-Opercular, opercular part of the inferior frontal gyrus; G_occipital_sup, superior occipital gyrus; G_oc-temp_med-Lingual, lingual gyrus, lingual part of the medial occipito-temporal gyrus; G_orbital, orbital gyri; SCN, structural covariance network; S_circular_insula_sup, superior segment of the circular sulcus of the insula; S_front_inf, inferior frontal sulcus; S_oc-temp_med_and_Lingual, medial occipito-temporal sulcus (collateral sulcus) and lingual sulcus; S_orbital_lateral, lateral orbital sulcus.

**Table S5.** **Results of Seed-based SCN analysis (Schaefer100_7)**

| Seed | Different covariance brain regions | *Z* score | *P* value |
| --- | --- | --- | --- |
| Left whole hippocampus | 7Networks_RH_Vis_2 | 4.701 | 0.0001 |
| Left presubiculum | 7Networks_LH_Default_PFC_2 | 4.626 | 0.0007 |
|  | 7Networks_RH_Vis_2 | 4.274 | 0.0001 |
|  | 7Networks_RH_DorsAttn_PrCv_1 | 4.763 | 0.0003 |
| Left subiculum | 7Networks_RH_Vis_2 | 3.951 | 0.0001 |
|  | 7Networks_RH_DorsAttn_PrCv_1 | 3.544 | 0.0051 |
| Left CA1 | 7Networks_RH_Vis_2 | 3.484 | 0.0035 |
| Left CA4 | 7Networks_RH_Vis_2 | 4.379 | 0.0001 |
|  | 7Networks_RH_Limbic_OFC_1 | 5.018 | 0.0129 |
| Left GC-DG | 7Networks_RH_Vis_2 | 4.587 | 0.0001 |
|  | 7Networks_RH_Limbic_OFC_1 | 4.941 | 0.0142 |
| Left molecular_layer_HP | 7Networks_RH_Vis_2 | 4.994 | 0.0001 |
| Right presubiculum | 7Networks_RH_Vis_7 | -3.739 | 0.0020 |
| Left whole amygdala | 7Networks_RH_Vis_2 | 4.150 | 0.0001 |
| Left corticoamygdaloid transition area | 7Networks_RH_Limbic_OFC_1 | 4.337 | 0.0311 |
| Left basal nucleus | 7Networks_RH_Vis_2 | 3.680 | 0.0014 |
| Left lateral nucleus | 7Networks_LH_SalVentAttn_FrOperIns_2 | 3.916 | 0.0388 |
|  | 7Networks_RH_Vis_2 | 3.935 | 0.0008 |
| Left accessory basal nucleus | 7Networks_RH_Vis_2 | 3.889 | 0.0028 |
| Right cortical nucleus | 7Networks_RH_Limbic_TempPole_1 | -4.630 | 0.0098 |
| Left caudate nucleus | 7Networks_LH_Cont_PFCl_1 | -3.970 | 0.0001 |
|  | 7Networks_RH_SalVentAttn_FrOperIns_1 | -4.451 | 0.0084 |

**Table S6. Results of Seed-based SCN analysis (Schaefer200_7)**

| Seed | Different covariance brain regions | *Z* score | *P* value |
| --- | --- | --- | --- |
| Left whole hippocampus | 7Networks_RH_Vis_4 | 4.701 | 0.0001 |
| Left presubiculum | 7Networks_LH_Cont_PFCl_1 | 4.626 | 0.0006 |
|  | 7Networks_RH_Vis_4 | 4.274 | 0.0003 |
|  | 7Networks_RH_DorsAttn_PrCv_1 | 4.763 | 0.0004 |
| Left subiculum | 7Networks_RH_Vis_2 | 3.951 | 0.0001 |
|  | 7Networks_RH_DorsAttn_PrCv_1 | 3.544 | 0.0027 |
| Left CA1 | 7Networks_RH_Vis_4 | 3.484 | 0.0037 |
| Left CA4 | 7Networks_RH_Vis_4 | 4.379 | 0.0002 |
|  | 7Networks_RH_Limbic_OFC_1 | 5.018 | 0.0106 |
| Left GC-DG | 7Networks_RH_Vis_2 | 4.587 | 0.0001 |
|  | 7Networks_RH_Limbic_OFC_1 | 4.941 | 0.0149 |
| Left molecular_layer_HP | 7Networks_RH_Vis_2 | 4.994 | 0.0001 |
| Right presubiculum | 7Networks_RH_Vis_15 | -3.739 | 0.0032 |
| Left whole amygdala | 7Networks_RH_Vis_4 | 4.150 | 0.0001 |
| Left corticoamygdaloid transition area | 7Networks_RH_Limbic_OFC_1 | 4.337 | 0.0335 |
| Left basal nucleus | 7Networks_RH_Vis_4 | 3.680 | 0.0018 |
| Left lateral nucleus | 7Networks_LH_SalVentAttn_FrOperIns_3 | 3.916 | 0.0371 |
|  | 7Networks_RH_Vis_2 | 3.935 | 0.0007 |
| Left accessory basal nucleus | 7Networks_RH_Vis_3 | 3.889 | 0.0016 |
| Right cortical nucleus | 7Networks_RH_Default_Temp_1 | -4.630 | 0.0087 |
| Left caudate nucleus | 7Networks_LH_Cont_PFCl_4 | -3.970 | 0.0001 |
|  | 7Networks_RH_SalVentAttn_FrOperIns_4 | -4.451 | 0.0106 |

**Table S7. Results of Seed-based SCN analysis (Schaefer400_7)**

| Seed | Different covariance brain regions | *Z* score | *P* value |
| --- | --- | --- | --- |
| Left whole hippocampus | 7Networks_RH_Vis_5 | 4.701 | 0.0001 |
| Left presubiculum | 7Networks_LH_Cont_PFCl_1 | 4.626 | 0.0004 |
|  | 7Networks_RH_Vis_12 | 4.274 | 0.0001 |
|  | 7Networks_RH_SalVentAttn_FrOperIns_8 | 4.763 | 0.0001 |
| Left subiculum | 7Networks_RH_Vis_5 | 3.951 | 0.0002 |
|  | 7Networks_RH_Cont_PFCl_7 | 3.544 | 0.0049 |
| Left CA1 | 7Networks_RH_Vis_5 | 3.484 | 0.0031 |
| Left CA4 | 7Networks_RH_Vis_5 | 4.379 | 0.0002 |
|  | 7Networks_RH_Limbic_OFC_3 | 5.018 | 0.0110 |
| Left GC-DG | 7Networks_RH_Vis_5 | 4.587 | 0.0001 |
|  | 7Networks_RH_Limbic_OFC_3 | 4.941 | 0.0164 |
| Left molecular_layer_HP | 7Networks_RH_Vis_5 | 4.994 | 0.0001 |
| Right presubiculum | 7Networks_RH_Vis_30 | -3.739 | 0.0025 |
| Left whole amygdala | 7Networks_RH_Vis_5 | 4.150 | 0.0001 |
| Left basal nucleus | 7Networks_RH_Vis_5 | 3.680 | 0.0016 |
| Left lateral nucleus | 7Networks_LH_SalVentAttn_FrOperIns_6 | 3.916 | 0.0378 |
|  | 7Networks_RH_Vis_5 | 3.935 | 0.0007 |
| Left accessory basal nucleus | 7Networks_RH_Vis_5 | 3.889 | 0.0032 |
| Left caudate nucleus | 7Networks_LH_Cont_PFCl_6 | -3.970 | 0.0001 |
|  | 7Networks_RH_SalVentAttn_FrOperIns_6 | -4.451 | 0.0097 |

**Supplementary Methods**

**Base dataset**

GWAS summary statistics from the first phase of the Genetics of Personality Consortium (GPC-1)[1] were used as the base dataset in polygenic score (PGS) analysis. The GPC is a large collaboration of genome-wide association studies (GWASs) consisting of two phases. GPC aims to identify genetic variants associated with personality and to further our understanding of the molecular genetic basis of personality traits. The GPC-1 combined results of 10 GWASs for each of the Big Five personality traits (Extraversion, Agreeableness, Conscientiousness, Neuroticism, and Openness to Experience), including 17 375 individuals of European ancestry from Europe, the United States, and Australia.

**Target dataset**

Alzheimer’s disease Neuroimaging Initiative (ADNI) database (https://adni.loni.usc.edu) was used as the target dataset. ADNI is a longitudinal multicenter study launched in 2003 designed to develop clinical, imaging, genetic, and biochemical biomarkers for the early detection and tracking of Alzheimer’s disease (AD). Normal cognition (NC) and mild cognitive impairment (MCI) subjects at baseline and with baseline and year 1 neuropsychological assessment from ADNI-1, ADNI-GO, and ADNI-2 cohorts (total *n* = 1208) were enrolled from the ADNI database (<https://adni.loni.usc.edu>) for the current study. The cognitive states of all participants were reclassified as NC or MCI at baseline and reclassified as NC, MCI, or dementia at year 1 based on Jak/Bondi neuropsychological (NP) criteria[2] and ADNI’s AD criteria[3]. The Jak/Bondi NP criteria included two memory measures: Rey Auditory Verbal Learning Test (AVLT) delayed free recall and AVLT recognition; two language measures: 30-item Boston Naming Test (BNT), animal fluency; and two attention/executive function measures: Trail Making Test (TMT), part A and part B. The scores of the tests were then converted to z-scores adjusted for age, sex, and education based on regression coefficients obtained from ADNI’s stable CN population (*n* = 373). Afterward, an MCI diagnosis would be made if a participant meets any of the following three criteria: 1) performance >1 SD below the age/sex/education-adjusted mean on both measures within any of three cognitive domains (i.e., language, memory, or attention/executive function); 2) performance >1 SD below the demographically adjusted mean on at least one measure in each of three cognitive domains; 3) has a Functional Activities Questionnaire (FAQ) score >5. Participants without dementia who did not meet NP criteria for MCI were considered CN. In total, 526 participants were reclassified as MCI at baseline, and 433 participants who remained stable (MCI-S, *n* = 343) or reverted to NC (MCI-R, *n* = 90) at year 1 were selected as target sample for the following analyses.

**Genotyping and Quality control (QC) for ADNI-1 and ADNI-GO/2**

Among 433 participants, 216 from the ADNI-1 cohort were genotyped using the Illumina Human610-Quad BeadChip, and 181 from ADNI-GO/2 cohorts were genotyped using the Illumina HumanOmniExpress BeadChip. For the genotype data from ADNI-1, we used the LiftOver tool in the UCSC Genome Browser (https://genome.ucsc.edu/cgi-bin/hgLiftOver)[4] to convert genome coordinates from NCBI36/hg18 to NCBI37/hg19. Afterward, the QC procedures were performed using the PLINK (version 1.90 beta6) (https://www.cog-genomics.org/plink2/)[5]. In the sample-level QC, subjects with a genotyping rate < 90%, possible relative relationship by using the estimate of pairwise identity-by-descent, and sex mismatch between genotyping and self-reported data were excluded. 1 participant in ADNI-1 and 1 participant in ADNI-GO/2 were excluded. In variant-level QC, SNPs with call rate < 85%, minor allele frequency (MAF) < 0.01, Hardy-Weinberg equilibrium (HWE) *P* < 1e-6, and ambiguous strands were excluded. After the sample- and variant-level QC, 215 individuals and 550 834 variants (genotyping rate of 99.51%) in ADNI-1 were included in the further imputation, and 180 individuals and 649 700 variants (genotyping rate of 99.83%) in ADNI-GO/2 were retained in the following imputation.

**Imputation and following QC**

The retained SNPs were pre-phased by SHAPEIT2[6] and imputed by IMPUTE2[7] with 1000 Genomes Phase 3 as reference panel. We retained SNPs with IMPUTE2 info quality score > 0.8, then merged ADNI-1 and ADNI-GO/2 genotype data. The merged genotype data was filtered with MAF > 0.05, call rate > 99%, and HWE *P* > 1e-6. Finally, 395 subjects (MCI-R, *n* = 85; MCI-S, *n* = 310) and 3 368 385 autosomal SNPs were included in the PGS calculation.

**DODS model**

A linear model implies two parameters: an offset (also called intercept) and a slope. “DODS” is a term unique to FreeSurfer, referring to “Different Offset, Different Slope”. In the current study, the offset/intercept is the cortical volume at volumes of subcortical structures of 0, and the slope is the rate of change of the cortical volume. The DODS model uses a separate linear model for each group (MCI-R or MCI-S), meaning that each group has its own offset and its own slope (thus DODS) for a total of four parameters: a. Regressor 1: has a value of 1 if the subject is a reverter, 0 otherwise. This regressor codes for membership in the MCI-R group. b. Regressor 2: has a value of 1 if the subject is a stable MCI participant, 0 otherwise. This regressor codes for membership in the MCI-S group. c. Regressor 3: has a value of the subject's subcortical structural volume if the subject is a reverter, 0 otherwise. This regressor codes for the slope of the reverters' cortical volume vs subcortical structural volume. d. Regressor 4: has a value of the subject's subcortical structural volume if the subject is a stable MCI participant, 0 otherwise. This regressor codes for the slope of the stable MCI participant s' cortical volume vs subcortical structural volume. The number of DODS regressors here is: NregressorsDODS = Nclasses * (Nvariables +1 ) = 2 * (1 + 1) = 4.

Here, we opted for the DODS model because we hypothesized that the MCI-R and MCI-S groups may exhibit distinct rates of cortical volume changes. By setting contrasts (0 0 1 -1), we tested whether there are differences in the slopes of cortical volumes between the two groups. These differences reflect the interaction between the two groups and subcortical structural volumes. The details of the DODS model can be found at the website <https://surfer.nmr.mgh.harvard.edu/fswiki/FreeSurferWiki>.

**References**

[1] M. H. de Moor, P. T. Costa, A. Terracciano, R. F. Krueger, E. J. de Geus, T. Toshiko, B. W. Penninx, T. Esko, P. A. Madden, J. Derringer, N. Amin, G. Willemsen, J. J. Hottenga, M. A. Distel, M. Uda, S. Sanna, P. Spinhoven, C. A. Hartman, P. Sullivan, A. Realo, J. Allik, A. C. Heath, M. L. Pergadia, A. Agrawal, P. Lin, R. Grucza, T. Nutile, M. Ciullo, D. Rujescu, I. Giegling, B. Konte, E. Widen, D. L. Cousminer, J. G. Eriksson, A. Palotie, L. Peltonen, M. Luciano, A. Tenesa, G. Davies, L. M. Lopez, N. K. Hansell, S. E. Medland, L. Ferrucci, D. Schlessinger, G. W. Montgomery, M. J. Wright, Y. S. Aulchenko, A. C. Janssens, B. A. Oostra, A. Metspalu, G. R. Abecasis, I. J. Deary, K. Räikkönen, L. J. Bierut, N. G. Martin, C. M. van Duijn, D. I. Boomsma, *Molecular psychiatry* **2012**, *17* (3), 337, https://doi.org/10.1038/mp.2010.128.

[2] K. R. Thomas, E. C. Edmonds, J. S. Eppig, C. G. Wong, A. J. Weigand, K. J. Bangen, A. J. Jak, L. Delano-Wood, D. R. Galasko, D. P. Salmon, S. D. Edland, M. W. Bondi, *Alzheimers Dement* **2019**, *15* (10), 1322, https://doi.org/10.1016/j.jalz.2019.06.4948.

[3] G. McKhann, D. Drachman, M. Folstein, R. Katzman, D. Price, E. M. Stadlan, *Neurology* **1984**, *34* (7), 939.

[4] A. S. Hinrichs, D. Karolchik, R. Baertsch, G. P. Barber, G. Bejerano, H. Clawson, M. Diekhans, T. S. Furey, R. A. Harte, F. Hsu, J. Hillman-Jackson, R. M. Kuhn, J. S. Pedersen, A. Pohl, B. J. Raney, K. R. Rosenbloom, A. Siepel, K. E. Smith, C. W. Sugnet, A. Sultan-Qurraie, D. J. Thomas, H. Trumbower, R. J. Weber, M. Weirauch, A. S. Zweig, D. Haussler, W. J. Kent, *Nucleic Acids Res* **2006**, *34* (Database issue), D590.

[5] S. Purcell, B. Neale, K. Todd-Brown, L. Thomas, M. A. R. Ferreira, D. Bender, J. Maller, P. Sklar, P. I. W. de Bakker, M. J. Daly, P. C. Sham, *Am J Hum Genet* **2007**, *81* (3), 559.

[6] O. Delaneau, J.-F. Zagury, J. Marchini, *Nat Methods* **2013**, *10* (1), 5, https://doi.org/10.1038/nmeth.2307.

[7] B. N. Howie, P. Donnelly, J. Marchini, *PLoS Genet* **2009**, *5* (6), e1000529, https://doi.org/10.1371/journal.pgen.1000529.
